# FGF4 and ascorbic acid enhance the maturation of induced cardiomyocytes by activating JAK2–STAT3 signaling

**DOI:** 10.1038/s12276-024-01321-z

**Published:** 2024-10-01

**Authors:** Seongmin Jun, Myeong-Hwa Song, Seung-Cheol Choi, Ji-Min Noh, Kyung Seob Kim, Jae Hyoung Park, Da Eun Yoon, Kyoungmi Kim, Minseok Kim, Sun Wook Hwang, Do-Sun Lim

**Affiliations:** 1 Department of Cardiology, Cardiovascular Center, College of Medicine, Korea University, Seoul, Republic of Korea; 2 R&D Center for Companion Diagnostic, SOL Bio Corporation, Seoul, Republic of Korea; 3 Department of Biomedical Sciences, College of Medicine, Korea University, Seoul, Republic of Korea; 4 Department of Physiology, College of Medicine, Korea University, Seoul, Republic of Korea

**Keywords:** Transdifferentiation, Reprogramming, Genetic vectors

## Abstract

Direct cardiac reprogramming represents a novel therapeutic strategy to convert non-cardiac cells such as fibroblasts into cardiomyocytes (CMs). This process involves essential transcription factors, such as *Mef2c, Gata4*, *Tbx5* (MGT), *MESP1*, and *MYOCD* (MGTMM). However, the small molecules responsible for inducing immature induced CMs (iCMs) and the signaling mechanisms driving their maturation remain elusive. Our study explored the effects of various small molecules on iCM induction and discovered that the combination of FGF4 and ascorbic acid (FA) enhances CM markers, exhibits organized sarcomere and T-tubule structures, and improves cardiac function. Transcriptome analysis emphasized the importance of ECM-integrin-focal adhesions and the upregulation of the JAK2–STAT3 and TGFB signaling pathways in FA-treated iCMs. Notably, JAK2–STAT3 knockdown affected TGFB signaling and the ECM and downregulated mature CM markers in FA-treated iCMs. Our findings underscore the critical role of the JAK2–STAT3 signaling pathway in activating TGFB signaling and ECM synthesis in directly reprogrammed CMs.

**Figure:**
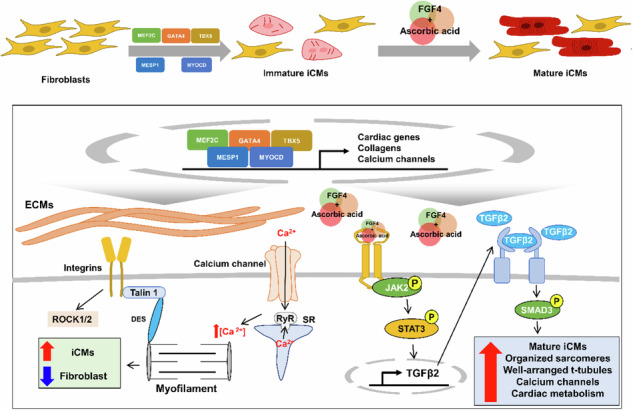
Schematic showing FA enhances direct cardiac reprogramming and JAK–STAT3 signaling pathways underlying cardiomyocyte maturation.

Schematic showing FA enhances direct cardiac reprogramming and JAK–STAT3 signaling pathways underlying cardiomyocyte maturation.

## Enhancing cardiac reprogramming: FGF4 and ascorbic acid synergy

Cardiovascular diseases are a major global cause of death, often due to the loss of cardiomyocytes and increased heart scarring. Existing treatments, like medication and heart transplants, have limitations, emphasizing the need for new cell regeneration therapies. This study investigates direct cardiac reprogramming—a new method to regenerate heart muscle cells by transforming fibroblasts into induced cardiomyocytes using specific factors and small molecules. The team tested various small molecules and found that a mix of FGF4 and ascorbic acid significantly improves the maturation of iCMs. They used techniques like immunofluorescence staining, flow cytometry, and electrophysiological analysis to evaluate the conversion and maturation of iCMs. This study shows that direct cardiac reprogramming can be enhanced with the right combination of small molecules, providing a promising strategy for heart regeneration.

This summary was initially drafted using artificial intelligence, then revised and fact-checked by the author.

## Introduction

Cardiovascular diseases (CVDs) are a significant and widespread health challenge contributing to elevated global mortality rates. CVDs encompass a range of conditions affecting heart and blood vessels, including coronary artery disease, heart failure, hypertension, and stroke. A major type of CVD is myocardial infarction, which results in cardiomyocyte (CM) loss and increased cardiac fibrosis. Patients with CVD typically exhibit CM death and dysfunction via the activation of fibroblasts/myofibroblasts due to myocardial dilation and decreased cardiac function^1–3^. Repair or regeneration of CMs is required for CVD recovery. However, human CMs have limited regenerative capacity. Although efforts to address the recovery of fibrotic CMs have advanced cardiovascular disease research and treatment, this remains a formidable global health issue that requires continuous research, prevention, and intervention strategies^3,4^.

In severe cases, traditional therapies for CVDs rely on drugs, lifestyle changes, and heart transplantation. However, the scarcity of donor hearts and the limitations of current treatments have spurred research into alternative strategies^5^. Therefore, only a few therapies are available for CVD regeneration or repair. Efforts to discover innovative technologies for heart regeneration, including pharmacological advancements and cell therapies, such as stem cell transplantation, are ongoing^6,7^. However, these efforts have shown limited effects, mainly because of the limited supply of cells for cardiac regeneration or repair in CVD patients and challenges associated with cellular mutations. Direct cardiac reprogramming is a promising therapeutic approach; it offers a method to regenerate and repair CMs by converting mature cells, such as fibroblasts, into induced CMs (iCMs) with transcription factors, microRNAs, and small molecules^8–12^. The fundamental principle of direct cardiac reprogramming involves converting somatic cells, typically fibroblasts or other non-cardiac cells, into induced CMs (iCMs) without an intermediate pluripotent state^13^. This direct conversion is achieved through the regulated manipulation of cellular factors and signaling pathways, and direct reprogramming has the advantage of reducing tissue damage while generating new CMs.

Direct cardiac reprogramming involves the overexpression of transcription factors that are upregulated in CMs. The screening of these transcription factors indicated that the combination of GATA4, MEF2C, and TBX5 (GMT) was optimal for reprogramming mouse fibroblasts^14^. Wang et al. reported that the combination of MEF2C, GATA4, and TBX5 (MGT) induces increased proportions of iCMs and beating cells^15^. In human fibroblasts, due to the inefficiency of inducing iCMs by GMT, a combination of GMT with MESP1 and MYOCD (GMTMM) has been used, resulting in significant changes in cell morphology toward a rod-like or polygonal shape and increased production of cardiac-specific proteins^16^. However, the overexpression of these transcription factors results in iCMs with a low transdifferentiation effect and immature characteristics^17,18^.

Various small molecules, including insulin-like growth factors (IGFs), triiodothyronine (T3), fibroblast growth factors (FGFs), and ascorbic acid (AA), have been reported to induce the differentiation of human pluripotent stem cells into mature CMs^19–23^. In direct cardiac reprogramming studies, inhibition of Notch signaling by DAPT increased the number of sarcomere+ and Ca^2+^ flux+ cells and beating iCMs^24^. SB431542, a transforming growth factor-beta (TGFB) inhibitor, also increased cardiac gene expression, organized sarcomeres, and decreased proliferation during direct cardiac reprogramming^25^. FGF2, FGF10, and vascular endothelial growth factor (VEGF) enhance quality of cardiac reprogramming by increasing the population of spontaneously beating iCMs^26^. However, studies on small molecules that induce mature iCMs have not been reported. Therefore, investigating small molecules related to the efficient conversion and maturation of iCMs is necessary. Furthermore, genes and biological signaling pathways related to the maturation of iCMs have recently been identified^27^.

In this study, we screened various small molecules, including chemicals, cytokines, and growth factors, and discovered that FGF4 and ascorbic acid (FA) induce direct cardiac reprogramming to produce mature iCMs. FA treatment enhanced the expression of cardiac channels and cardiac metabolic markers. Moreover, we identified genes and signaling pathways regulating fibroblast-to-iCM conversion and iCM maturation by transcriptome analysis and gene knockdown studies. Our findings show that understanding direct cardiac reprogramming and its potential to transform fibroblasts into functional CM holds promise as a novel approach for treating CVDs. This emerging field of research offers new avenues for developing regenerative therapies and treatments that can revolutionize the treatment of cardiovascular diseases.

## Materials and methods

### Immunofluorescence staining

At week 4 of reprogramming, mouse or human iCMs were reseeded onto gelatin-coated slides. The cells were fixed with 4% paraformaldehyde (PFA; Sigma-Aldrich) in PBS for 20 min at room temperature and permeabilized with 0.2% Triton X-100 in PBS for 30 min. The slides were washed three times with PBS containing 0.1% Tween 20 (PBST) and blocked with 5% normal goat serum (NGS; Thermo Fisher Scientific) in PBST for 1 h at room temperature. The blocked slides were incubated at 4 °C overnight with the following primary antibodies in 5% NGS in PBST: anti-ACTN, anti-TNNI3, anti-TNNT2, and anti-JPH2. The slides were then washed three times with PBST and incubated for 2 h at room temperature with the following secondary antibodies: Alexa Fluor 594-conjugated goat anti-mouse IgG, Alexa Fluor 647-conjugated goat anti-mouse IgG, and Alexa Fluor 555-conjugated goat anti-rabbit IgG. Nuclei were stained with 1 μg/mL DAPI. The slides were then mounted with fluorescent mounting medium (S3023, DAKO, Glostrup, Denmark). Immunofluorescence images were acquired with a fluorescence microscope and a confocal fluorescence microscope (LSM900 and LSM800; Carl Zeiss, Oberkochen, Germany). The primary and secondary antibodies used are listed in Supplementary Table 4. The intensity of the fluorescence images was quantified by ImageJ software (NIH, USA).

### Flow cytometry

Single cells were isolated with T/E buffer, fixed with 4% PFA in PBS for 20 min, and washed three times with PBS containing 2% FBS. The cells were then permeabilized with 100% ice-cold methanol for 20 min on ice. The cells were incubated for 45 min at RT with the primary antibody against TNNT2 (1:100; MA5-12960, Thermo Fisher Scientific) and S100A4 (1:100; 07-2274, Millipore, USA). The cells were then washed three times with PBS containing 2% FBS, followed by incubation with the corresponding secondary antibodies, goat anti-mouse IgG Alexa 647 (1:1000; A32728, Thermo Fisher Scientific) and goat anti-rabbit IgG Alexa 647 (1:1000; A21244, Invitrogen), for 20 min. The cells were run through a BD FACSCanto™ II flow cytometer (BD Biosciences) and analyzed by FlowJo software.

### Western blot analysis

The cells were washed with PBS and lysed with ice-cold 1× cell lysis buffer (9803; Cell Signaling Technology, Danvers, MA, USA) containing 1 mM phenylmethylsulfonyl fluoride (P7626; Sigma-Aldrich). Protein concentrations were quantified with the Bradford assay dye reagent (500-0006, Bio-Rad Laboratories). Protein samples (15–20 μg) mixed with 1× loading dye were boiled for 8 min. The proteins were separated by electrophoresis on a 10% sodium dodecyl sulfate–polyacrylamide gel and transferred to a polyvinylidene fluoride membrane (10600023; Thermo Fisher Scientific). The membranes were blocked with 5% BSA in Tris-buffered saline containing 0.1% Tween 20 (TBST) for 1 h at room temperature. The membranes were incubated overnight at 4 °C with primary antibodies. The primary antibodies used for western blot analysis are listed in Supplementary Table 4. The membranes were washed three times with TBST and incubated with a horseradish peroxidase-conjugated secondary antibody in TBST at room temperature for 1 h. The bands were visualized with ECL (32106, Thermo Fisher Scientific, USA), ECL Plus reagent (32132, Thermo Fisher Scientific), and West Femto reagent (34095, Thermo Scientific) by the ChemiDoc^TM^ Touch Imaging System (Bio-Rad Laboratories) using the enhanced chemiluminescence detection system. The signal intensity was analyzed by Image Lab Software (Bio-Rad Laboratories).

### Electrophysiological analysis

For electrophysiological analysis, at week 4 of reprogramming, mouse iCMs were seeded on glass cover slips. The iCMs were superfused with a bath solution containing 140 mM NaCl, 5 mM KCl, 2 mM CaCl_2_, 1 mM MgCl, 10 mM HEPES, and 10 mM d-glucose with a pH of 7.2–7.3 adjusted with NaOH and an osmolarity of 310–320 mOsm/kg. For measurements of the AP, a current clamp was used in the whole-cell configuration. Borosilicate glass pipettes (Sutter Instrument, Novato, CA, USA) with a resistance of 3–6 MΩ were filled with pipette solution containing 110 mM K-gluconate, 25 mM KCl, 1 mM CaCl_2_, 4 mM MgATP, 0.5 mM Na_2_GTP, 10 mM HEPES, and 10 mM EGTA with a pH of 7.28 adjusted with NaOH and an osmolarity of 290–305 mOsm/kg. The data were acquired by Patch Master software (HEKA instruments) with an EPC-10 USB patch clamp amplifier. The data were low-pass filtered at 2 kHz and digitized at 5 kHz.

### RNA-seq analysis

Samples were collected at week 4 of direct reprogramming for RNA-seq analysis. Total RNA was extracted using TRIzol reagent following the manufacturer’s instructions, and the RNA concentration and quality were assessed with a NanoDrop spectrophotometer and an Agilent 2100 bioanalyzer with an RNA 6000 Nano Chip (Agilent Technologies, Amstelveen, The Netherlands). Libraries for RNA-seq analysis were subsequently prepared with the QuantSeq Library Prep Kit (Lexogen Inc., Vienna, Austria). High-throughput sequencing was performed with a NextSeq 500 (Illumina, Inc., San Diego, CA, USA). We compared the whole transcriptomes and identified differentially expressed genes (DEGs) that exhibited more than twofold changes in expression. DEGs were analyzed by ExDEGA software (EBIOGEN, Inc., Seoul, Korea). For gene classification, searches were performed with the DAVID (http://david.abcc.ncifcrf.gov/), Gene Ontology (http://www.ebi.ac.uk/QuickGO/), Kyoto Encyclopedia of Genes and Genomes (KEGG) pathway (http://www.genome.jp/kegg/tool/map_pathway2.html), and STRING (http://www.string-db.org/) databases.

### Statistical analyses

All experiments were performed with at least three independent experimental sets, and the results are presented as the means ± standard deviations. Statistical analysis was performed with Student’s *t*-test for two-group comparisons and ANOVA for multiple-group comparisons to determine the significance of the differences. The data were analyzed by GraphPad Prism version 8.0.2. (GraphPad Software, Inc., San Diego, CA, USA).

## Results

### Screening for maturation factors to enhance the conversion of mature CMs

**Fig. 1:**
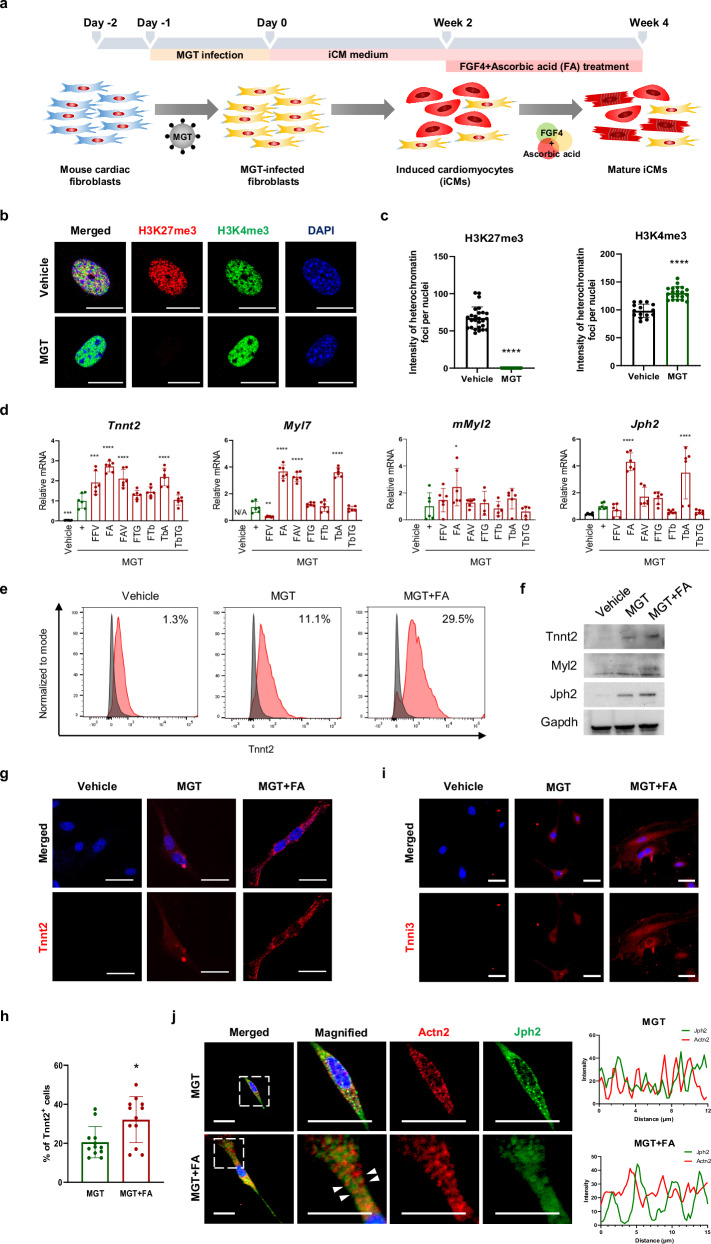
FA significantly promotes the conversion of partially reprogrammed iCMs into mature iCMs. **a** Schematic representation of the strategy and protocol for testing candidate maturation factors affecting direct cardiac reprogramming. **b** Dual immunofluorescence staining depicting the levels of the histone methylation markers H3K4me3 (green) and H3K27me3 (red) in vehicle- and MGT-transfected cells at week 1. Scale bars = 50 μm. Nuclei were stained with DAPI (blue). **c** Quantification of the fluorescence intensity of H3K4me3 and H3K27me3 in the nuclei of MGT-transfected cells compared with vehicle control cells (*n* = 14). **d** mRNA expression of a total CM marker (*Tnnt2*), an atrial CM marker (*Myl7*), a ventricular CM marker (*Myl2*), and a T-tubule marker (*Jph2*) determined by qPCR in iCMs at week 4 (*n* = 6). **e** FACS analysis of Tnnt2 expression in the vehicle, MGT, and MGT + FA at week 4 after MGT transfection. **f** Western blot of Tnnt2, Myl2, and Jph2 normalized to GAPDH (*n* = 3). **g** Immunofluorescence staining for Tnnt2 (red) and nuclei (DAPI) (blue) in the vehicle, MGT and MGT + FA. **h** Quantitative data of Tnnt2*-*immunopositive MGT- and MGT + FA-treated cells at week 4 (*n* = 12). **i** iCMs showing Tnni3 (red) and nuclei (DAPI) (blue) immunofluorescence at week 4 after transfection. **j** Representative immunofluorescence staining for the cardiac sarcomere markers *Actn* (red) and *Jph2* (green) and nuclei (DAPI) (blue) at week 4 after MGT transfection. Scale bars = 50 μm (**g**, **i**, and **j**). All the data are presented as the means ± SDs. ****p < 0.0001 versus the vehicle (**c**) and *p < 0.05, **p < 0.01, ***p < 0.001, ****p < 0.0001 versus the MGT (**d** and **h**).

To obtain mature CMs from mouse embryonic fibroblasts (MEFs) through direct cardiac reprogramming, we first optimized the protocol by overexpressing three cardiac transcription factors (*Mef2c*, *Gata4*, and *Tbx5*; MGT) and then screened various combinations of growth factors^28^. MEFs were infected with retroviruses encoding MGT genes and transferred into iCM medium containing puromycin to select infected cells (Fig. 1a). After week 2, we attempted to differentiate them into mature CMs by adding the test factors to the maturation medium (StemPro-34) for an additional 2 weeks^26^ (Fig. 1a).

Direct cardiac reprogramming is regulated by epigenetic modifications such as histone H3 lysine residue position 4 (H3K4me3) and histone H3 lysine 27 (H3K27me3). H3K27me3 silences fibroblast-specific genes and induces the expression of cardiac-specific genes, whereas H3K4me3 activates cardiac-specific genes during direct mouse cardiac reprogramming^29^. To examine the remodeling of chromatin architecture, we analyzed the levels of H3K27me3 and H3K4me3 by immunofluorescence staining at week 1. Compared with those in the vehicle group, H3K27me3 was weakly detected and H3K4me3 was strongly detected in the MGT group (Fig. 1b, c).

To investigate the combination of maturation factors that promote functional iCMs, we used various small molecules, including FGF2, FGF4, FGF10, ascorbic acid (AA), VEGF, TGFB1, and 1-thioglycerol (1-TG). We performed qRT-PCR for a total CM marker (*Tnnt2*), an atrial CM marker (*Myl7*), a ventricular CM marker (*Myl2*), and a T-tubule marker (*Jph2*) to determine whether maturation factors induced iCM maturation (Fig. 1d). We used FFV growth factors (FGF2, FGF10, and VEGF) as positive controls^26^. Compared with MGT, the combination of FGF4 + AA (FA) and TGFB + AA (TbA) significantly increased the levels of *Tnnt2*, *Myl7*, and *Jph2*, while *Myl2* was higher only in the MGT + FA group. Compared with MGT, the positive control, MGT + FFV, resulted in decreased levels of Myl7. To enhance reprogramming efficiency, we explored the best combination, FA, with/without small molecules such as TGFB1 and 1-TG (Supplementary Fig. 1). Compared with those in the MGT group, Tnnt2, Myl7, and Jph2 levels were increased in the MGT + FA, MGT + FATb, and MGT + FATG groups. Interestingly, *Myl2* was significantly increased only in MGT + FA compared to that in MGT. Overall, these results demonstrate that the combination of FGF4 and AA enhanced the efficiency of direct cardiac reprogramming in MEFs.

Next, we investigated the optimal timing for reprogramming MEFs into mature iCMs with FA by changing the timing of the FA treatment to weeks 1–2, 2–4, and 1–4, respectively, after MGT transfection. Expression of *Tnnt2* showed a fourfold increase after FA treatment between 2 and 4 weeks compared with MGT treatment, although FA treatment for the other periods only slightly affected direct reprogramming (Supplementary Fig. 2a). qRT-PCR analysis revealed that the cardiac-specific maturation markers *Mly7*, *Myl2, Cav3*, and *Jph2* were significantly increased after 2–4 weeks compared with those in the MGT (Supplementary Fig. 2b). These results suggest that FA enhanced the maturation of iCMs between 2 and 4 weeks of reprogramming and played a crucial role in reprogramming MEFs into mature iCMs.

We also investigated the proportions of iCMs in the vehicle, MGT, and MGT + FA by flow cytometry at weeks 2 and 4. At week 2, the percentage of TnnT2^+^ iCMs was 7% in the MGT. By week 4, this percentage had increased to 11.1% in the MGT group and 29.5% in the MGT + FA group compared with that in the vehicle group (1.3%) (Fig. 1e). Additionally, the expression of the fibroblast marker S100a4 was 93.2% in the vehicle and 90% in the MGT at week 2. By week 4, the proportions were 91.3% in the vehicle, 88.1% in the MGT, and a significantly reduced 61.7% in the MGT + FA (Supplementary Fig. 3). We subsequently assessed the expression of Tnnt2, *Myl7, Myl2*, and *Jph2* by western blotting (Fig. 1f). The levels of *Tnnt2, Myl7, Myl2*, and *Jph2* were higher in the MGT group than in the vehicle group and were further enhanced by FA treatment. Immunofluorescence staining for *Tnnt2* and the mature CM marker *Tnni3* was performed to examine morphology and cardiac structure (Fig. 1g, i). *Tnnt2* and *Tnni3* were expressed in the MGT and MGT + FA but not in the vehicle. Compared with the MGT group, in the MGT + FA group, there was a larger number of Tnnt2^+^ and Tnni3^+^ iCMs and a stronger expression of these markers. The percentage of *Tnnt2*-positive cells was ~30% in the MGT + FA group and 20% in the MGT group (Fig. 1h).

To examine the conversion of fibroblasts into structurally mature iCMs, we stained for the cardiac sarcomere markers Actn2 and Jph2 in the MGT and MGT + FA groups. Actn2 and Jph2 were co-expressed in the MGT + FA, and their sarcomeres and T-tubules were regularly arranged compared to those in the MGT. According to the fluorescence intensity analysis, the Actn2 signal showed an alternating pattern with the Jph2 signal (Fig. 1j). The percentage of Tnnt2/Jph2-double-positive cells was ~1.3 times greater in the MGT + FA than in the MGT (data not shown). These results indicate that the combination of FGF4 and AA induced the formation of structurally mature iCMs from immature MEFs.

### FA-treated iCMs show elevated expression of ion channel genes

**Fig. 2:**
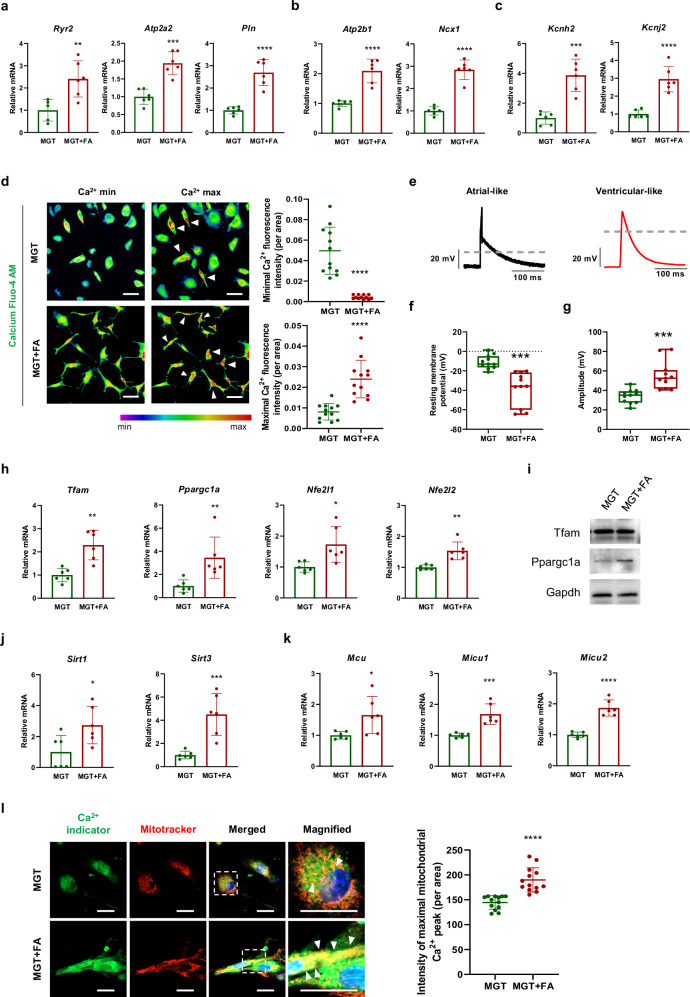
MGT + FA substantially increases the expression of calcium channel-related genes and enhances mitochondrial calcium exchange. mRNA expression of **a** an L-type Ca^2+^-related marker (*Ryr2*), Ca^2+^ ATPase markers (*Atp2b1* and *Pln*), **b** a plasma membrane calcium pump marker (*Atp2b1*), and a Na^+^Ca^2+^ exchanger marker (*Ncx1*) in the MGT and MGT + FA by qRT-PCR (*n* = 6). **c** mRNA expression of K+ channel markers analyzed by qRT-PCR (*n* = 6). **d** Spontaneous Ca^2+^ oscillations observed in iCMs treated with Fluo-4AM at the Ca^2+^ maximum and minimum. Quantification of the maximum and minimum Ca^2+^ intensities in randomly selected fields (*n* = 12, scale bars = 20 μm). **e** Representative images of electrical stimulation-induced atrial-like and ventricular-like APs. **f** Resting membrane potential was measured in the MGT (*n* = 11) and MGT + FA (*n* = 11). **g** Amplitude was measured in the MGT (*n* = 11) and MGT + FA (*n* = 11). **h** Relative mRNA expression of mitochondrial biogenesis markers in iCMs determined by qRT‒PCR (*n* = 6). **i** Western blotting of *Tfam* and *Ppargc1a* expression in MGT-infected MEFs. The expression levels were normalized to *Gapdh*. **j** mRNA expression of mitochondrial ROS markers (*Sirt1* and *Sirt3*) and **k** mitochondrial calcium uniporter channel markers (*Mcu*, *Micu1*, and *Micu2*) determined by qRT-PCR (*n* = 6). **l** Representative fluorescence images of the calcium indicator and mitochondria detection marker in the MGT and MGT + FA. Merged images indicate mitochondrial Ca^2+^ accumulation (white arrow; scale bars = 20 μm). The intensity of maximal mitochondrial Ca^2+^ flux was quantified in the MGT and MGT + FA groups (*n* = 13). All the data are presented as the means ± SDs. *p < 0.05, **p < 0.01, ***p < 0.001, ****p < 0.0001 versus the MGT.

Cardiac functions, including rhythmic contraction, electrical activity, and activity of ion channels, such as sodium, potassium, and Ca^2+^ channels, generate and regulate electrical impulses that drive heartbeats^30,31^. To evaluate the effects of FA on the generation of functionally mature iCMs from fibroblasts, we analyzed the expression of L-type Ca^2+^ channel markers (*Ryr2)*, Ca^2+^ ATPase markers (*Atp2b1* and *Pln*), a plasma membrane Ca^2+^ pump (*Atp2b1)*, a Na+–Ca^2+^ exchanger marker (*Ncx1*), and potassium channel markers (*Kcnj2* and *Kcnh2*) by qRT-PCR (Fig. 2a–c). The expression levels of *Ryr2*, *Atp2b1*, and *Pln* were significantly greater in the MGT + FA group than in the MGT group (Fig. 2a). The mRNA levels of *Atp2b1* and *Ncx1* also increased approximately twofold in the MGT + FA group compared with those in the MGT group (Fig. 2b). In mature ventricular CMs, potassium channels play an important role in the repolarization phase of action potentials^32^. qRT-PCR analysis revealed that the potassium channel markers *Kcnj2*, a potassium inwardly rectifying channel, and *Kcnh2*, a potassium voltage-gated channel, were significantly upregulated in the MGT + FA compared with the MGT (Fig. 2c). Furthermore, to analyze the intracellular Ca^2+^ flux in the MGT and MGT + FA at week 4, we used Fluo-4AM staining, in which bright green fluorescence directly correlates with the level of intracellular Ca^2+^ present. To quantify the Ca^2+^ level of the MGT + FA group compared with that of the MGT group, we determined the fluorescence intensity; treatment with FA triggered maximal Ca^2+^ oscillation, which lasted nearly 1 min, whereas rare Ca^2+^ oscillations were found in the MGT group (Fig. 2d). Furthermore, an electrophysiological analysis was performed to evaluate the enhancement of CM maturation by FA treatment. Representative images of ventricular- and arterial-like CMs are shown in Fig. 2e. The resting membrane potential in the MGT + FA was approximately −37.68 ± 26.75 mV, whereas it was −10.9 ± 12.4 mV in the MGT (Fig. 2f). The amplitude was 55.5 ± 26.6 mV in the MGT + FA, which was significantly greater than the 34.0 ± 12.4 mV in the MGT (Fig. 2g). These findings suggest that the combination of FGF4 and AA enhanced cardiac function by promoting cardiac ion channel function during the direct cardiac reprogramming of MEFs.

### FA increases mitochondrial biogenesis and ROS production and activates mitochondrial Ca^2+^

In CMs, mitochondria perform major biological processes, including metabolic regulation, Ca^2+^ handling, and redox generation. To explore the effects of FA treatment on mitochondrial biogenesis^33^, we evaluated the expression of mitochondrial biogenesis markers (*Tfam1*, *Ppargc1a*, *Nfe2l1*, and *Nfe2l2*) by qRT-PCR. *Tfam1*, *Ppargc1a*, *Nfe2l1*, and *Nfe2l2* were elevated in the MGT + FA compared with the MGT (Fig. 2h). Furthermore, the mRNA levels of mitochondrial metabolism and ROS markers (*Sirt1 and Sirt3*) were significantly greater in the MGT + FA than in the MGT (Fig. 2j). We further examined the protein levels of mitochondrial biogenesis markers by western blotting. Ppargc1a but not Tfam was more strongly detected in the MGT + FA than in the MGT group (Fig. 2i). Ca^2+^ regulates mitochondrial function in energy production for cardiac activity^34,35^. Therefore, to evaluate the function of cardiac contraction through Ca^2+^ regulation in mitochondria, we examined the expression of mitochondrial Ca^2+^ uniporter channel markers (*Mcu*, *Micu1*, and *Micu2*) by qRT-PCR. *Mcu*, *Micu1*, and *Micu2* were upregulated in the MGT + FA group compared with the MGT group (Fig. 2k). In addition, we used Ca^2+^ transient markers (Fluo-4AM) and mitochondrial markers (MitoTracker) to evaluate mitochondrial structure and function in Ca^2+^ uptake. The mitochondria in immature CMs are small and widely distributed in the cytoplasm and are present in the perinuclear region, whereas mature CMs exhibit larger mitochondria that are well-organized and located primarily within the intermyofibrillar or subsarcolemmal region^36,37^. Our results show that treating iCMs with FA increased the mitochondrial content and arrangement of cristae. Furthermore, upon merging the two indicators (Fluo-4AM and MitoTracker), a distinct overlap, denoted by the yellow fluorescence, was observed in the MGT + FA group, whereas there was no overlap between the distributions of Fluo-4AM and MitoTracker in the MGT group. This result indicates that mitochondrial Ca^2+^ function was observed in the mature CMs (white arrow in Fig. 2l). These results demonstrate that the overexpression of MGT transcription factors could induce the differentiation of fibroblasts into CMs through cell conversion and that treatment with FA could induce high maturation and conversion rates in iCMs.

### FA enhances the transdifferentiation efficiency in adult mouse cardiac fibroblasts

**Fig. 3:**
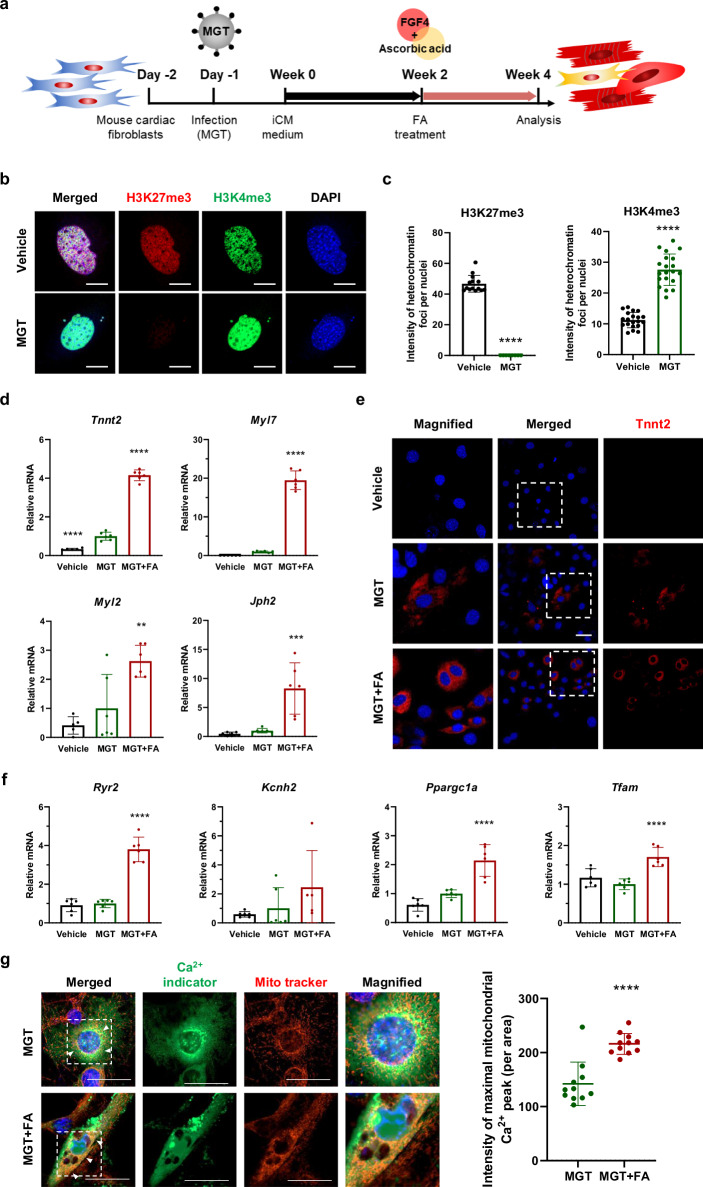
FA treatment increases direct cardiac reprogramming and induces the structural and metabolic maturation of iCMs from MCFs. **a** Schematic representation of the direct cardiac reprogramming experimental design from MCFs. **b** Immunofluorescence staining of *H3K27me3* (red), *H3K4me3* (green), and nuclei (DAPI) (blue) in vehicle- and MGT-transfected MCFs. Scale bars = 50 μm. **c** Quantitative fluorescence intensity analysis of H3K27me3 and H3K4me3 per nucleus (*n* = 20). **d** qRT-PCR of *Tnnt2*, *Myl7*, *Myl2*, and Jph2 in the vehicle, MGT, and MGT + FA groups at week 4. **e** Immunofluorescence staining for *Tnnt2* in iCMs at week 4. Scale bars = 20 μm. **f** Relative mRNA expression of *Ryr2*, *Kcnh2, Ppargc1a*, and *Tfam* in MGT-transfected MCFs determined by qRT-PCR (*n* = 6). **g** Representative fluorescence of Fluo-4AM and MitoTracker in the MGT and MGT + FA. Merged images indicate mitochondrial Ca^2+^ accumulation (white arrow). Maximal mitochondrial Ca^2+^ flux quantified in the MGT and MGT + FA (*n* = 11). Scale bars = 50 μm. All the data are presented as the means ± SDs. **p < 0.01, ***p < 0.001, ****p < 0.0001 versus the MGT.

The transfection of MGT into MEFs has been demonstrated to directly reprogram fibroblasts into iCMs without regression to a stem/progenitor state. According to other studies, the efficiency of cardiac reprogramming in adult cardiac fibroblasts (MCFs) is lower than that in MEFs^17,38^. Thus, we tested the effect of FA on MCFs to determine whether it directly converts fibroblasts into CM-like cells. A schematic representation of the experimental design is shown in Fig. 3a. To investigate chromatin architecture remodeling, we conducted immunofluorescence staining to examine the levels of H3K27me3 and H3K4me3 at week 1. Compared with the vehicle group, the MGT group had weak levels of H3K27me3 and strong levels of H3K4me3 (Fig. 3b, c). We performed qRT-PCR to evaluate the mRNA expression of cardiac-related markers, including *Tnnt2, Myl7, Myl2*, and *Jph2*. These results demonstrated that, compared with MGT treatment, FA treatment upregulated *Tnnt2, Myl7*, *and Jph2* but slightly affected *Myl2* (Fig. 3d). Next, we confirmed that FA improved the structural maturation of CMs by immunofluorescence staining for the cardiac marker *Tnnt2*. The MGT + FA group demonstrated a greater quantity of MGT-infected cells stained with Tnnt2, unlike the MGT and vehicle groups, where such expression was not observed (Fig. 3e). To determine whether iCMs exhibit functional characteristics typical of CMs, we analyzed *Ryr2* and *Kcnh2*, along with the genes *Ppargc1a* and *Tfam*, by qRT-PCR. The mRNA expression of these genes significantly increased in the MGT + FA group, but that of Kcnh2 was not significantly different (Fig. 3f). Finally, we used the Fluo-4AM marker and the mitochondrial marker MitoTracker to assess the mitochondrial Ca^2+^ (yellow; white arrow in Fig. 3g). We quantified the expression of mitochondrial Ca^2+^ when it peaked maximally and detected a significant increase in the MGT + FA compared with the MGT (Fig. 3g). These results indicate that the combination of FA improved direct cardiac reprogramming efficiency and successfully converted fibroblasts into functional CMs, even adult cardiac fibroblasts, which are known for their lower reprogramming efficiency.

### FA enhances the efficiency of cardiac reprogramming of human fibroblasts to iCMs

**Fig. 4:**
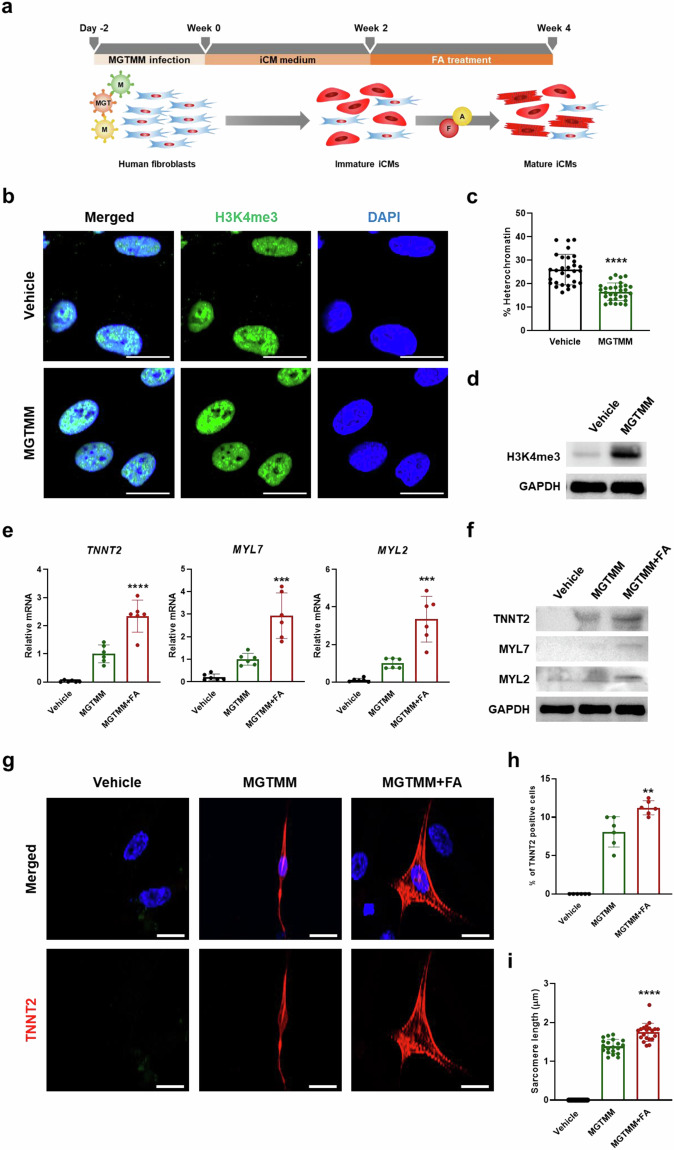
FA significantly enhances the efficiency of transdifferentiating fibroblasts into iCMs. **a** Schematic diagram showing the protocol of direct cardiac reprogramming of NHDFs into iCMs. **b** Immunofluorescence staining analysis of a histone methylation marker, H3K4me3 (green), in the vehicle and MGTMM at week 1. Scale bars = 20 μm. Nuclei were stained with DAPI (blue). **c** Quantification of the heterochromatin percentage in the vehicle and MGTMM. Values are means ± SDs. *n* = 30 for each group. ****p < 0.0001 versus the vehicle. **d** Western blotting of the histone methylation marker H3K4me3 in the vehicle and MGTMM. GAPDH served as a loading control. **e** qRT-PCR analysis of a total CM marker (*TNNT2*), an atrial CM marker (*MYL7*), and a ventricular CM marker (*MYL2*) in the vehicle, MGTMM, and MGTMM + FA at week 4. The values represent the means ± SDs. *n* = 6 for each group. ***p < 0.001, ****p < 0.0001 versus the MGTMM. **f** Western blotting of TNNT2 and MYL2 in the vehicle, MGTMM, and MGTMM + FA at week 4. GAPDH served as a loading control. **g** Immunofluorescence analysis of a total CM marker, TNNT2 (red), in the vehicle, MGTMM, and MGTMM + FA. Scale bars = 20 μm. Nuclei were stained with DAPI (blue). Quantification of **h** TNNT2-positive cells and **i** sarcomere length in the vehicle, MGTMM, and MGTMM + FA. Values are means ± SDs. *n* = 6 in (**h**) and *n* = 20 in (**i**) for each group. **p < 0.01, ****p < 0.0001 versus the MGTMM.

To investigate the effects of FA on cardiac induction in human fibroblasts, we optimized a direct cardiac reprogramming protocol using transcription factors and FA. In humans, MGT, MESP1, and MYOCD (MGTMM) increase the efficiency of iCM reprogramming, promoting the conversion of fibroblasts into iCMs^15,16^. We transduced normal human dermal fibroblasts (NHDFs) with three lentiviruses encoding five-core cardiac-specific factors (MGTMM; Fig. 4a and Supplementary Fig. 4a–c). We first investigated the efficiency of the MGT, MESP1, and MYOCD vectors by qRT-PCR in 293T cells (Supplementary Fig. 4d). *MEF2C*, *GATA4*, and *TBX5* were highly expressed in MGT vector-transfected 293T cells, *MESP1* was significantly upregulated in MESP1 vector-transfected 293T cells, and *MYOCD* was upregulated in MYOCD vector-transfected 293T cells. Furthermore, we treated cells with FA at week 2 and analyzed them at week 4 (Fig. 4a). Distinct morphological changes were observed in each group, and the levels of *GATA4*, *MEF2C*, *TBX5*, *MESP1*, and *MYOCD* were significantly increased in the MGTMM and MGTMM + FA compared to vehicle (Supplementary Fig. 5).

To evaluate the effect of MGTMM overexpression on reprogramming, we examined the expression levels of H3K4me3 by immunofluorescence staining on Day 7 (Fig. 4b). H3K4me3 was more strongly expressed in the MGTMM than in the vehicle. The percentage of H3K4me3 area relative to total nuclei decreased in the MGTMM group compared with the vehicle group (Fig. 4c). The protein level of H3K4me3 was also greater in the MGTMM than in the vehicle (Fig. 4d).

We examined the effects of the FA combination on the expression levels of *TNNT2*, *MYL7*, and *MYL2* by qRT-PCR and western blot analyses (Fig. 4e, f). The mRNA levels of *TNNT2*, *MYL7*, and *MYL2* were significantly higher in the MGTMM + FA than in the MGTMM (Fig. 4e). The protein levels of TNNT2, MYL7, and MYL2 were also greater in the MGTMM + FA group than in the MGTMM group (Fig. 4f). Interestingly, the nodal CM marker *TBX18* revealed no significant differences among the vehicle, MGTMM, and MGTMM + FA groups (Supplementary Fig. 6a). Next, we investigated the proportions of TNNT2^+^ iCMs in the vehicle, MGTMM, and MGTMM + FA by immunofluorescence staining (Fig. 4g). A large proportion of TNNT2^+^ iCMs (8.1%) and long sarcomere structures (1.2 μm) were more frequently observed in the MGTMM + FA than in the MGTMM (11.2% and 1.7 μm, respectively; Fig. 4h, i). We further assessed different cardiac markers (*TNNI3*, *MYH6*, and *MYH7*) and T-tubule markers (*BIN1*, *CAV3*, and *JPH2*) by qRT-PCR (Supplementary Fig. 6b, c). *TNNI3*, *MYH6*, *MYH7*, *BIN1*, *CAV3*, and *JHP2* mRNA expression was markedly increased in the MGTMM + FA compared with the MGTMM. Higher expression of CAV3 was also observed in the MGTMM + FA group than in the MGTMM group as determined by western blotting (Supplementary Fig. 6d). To investigate the ultrastructural array between T-tubules and sarcomeres, JPH2 and TNNT2 were examined by immunofluorescence staining. Analysis of well-organized T-tubules and sarcomeres revealed that areas in which JPH2 and TNNT2 were colocalized were observed more frequently in the MGTMM + FA than in the MGTMM (Supplementary Fig. 7a). Furthermore, examining the expression patterns of ACTN2 and JPH2 to analyze the cross-structures of T-tubules and Z-lines revealed a greater arrangement of cross-structures in the MGTMM + FA than in the MGTMM (Supplementary Fig. 7b, c). These results demonstrate that FA treatment enhanced cardiac gene expression at the reprogramming stage, indicating the potential of FA to increase the efficiency of direct cardiac reprogramming of human fibroblasts.

### FA improves iCM functions by enhancing Ca^2+^ channels and metabolism

**Fig. 5:**
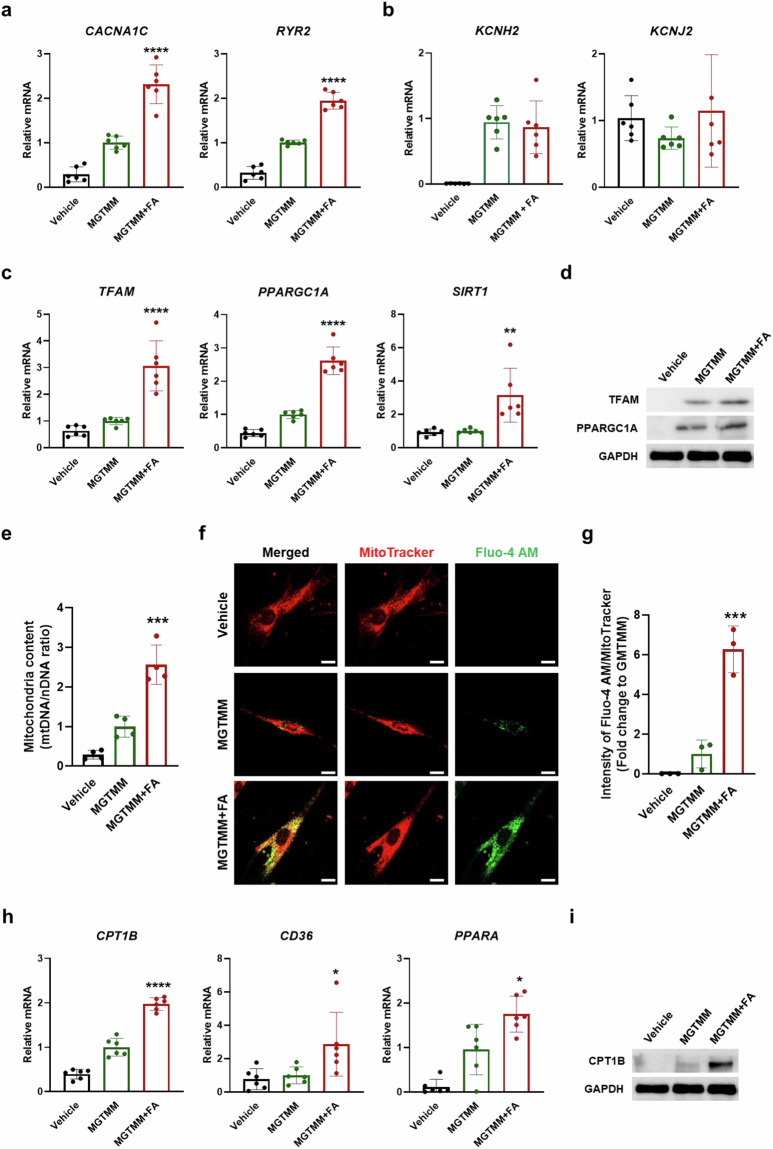
MGTMM + FA significantly enhances cardiac function in human iCMs. qRT-PCR showing the mRNA expression of **a** calcium channel markers (*CACNA1C* and *RYR2*), **b** potassium markers (*KCNH2* and *KCNJ2*), and **c** mitochondrial biogenesis markers (*TFAM*, *PPARGC1A*, and *SIRT1*) in the vehicle, MGTMM, and MGTMM + FA. Values represent the means ± SDs. *n* = 6 for each group. *p < 0.05, **p < 0.01, ****p < 0.0001 versus the MGTMM. **d** Western blotting of TFAM and PPARGC1A in the vehicle, MGTMM, and MGTMM + FA. GAPDH served as a loading control. **e** qRT-PCR showing the relative mtDNA copy numbers in the vehicle, MGTMM, and MGTMM + FA. Values represent the means ± SDs. *n* = 4 for each group. ***p < 0.001 versus the MGTMM. **f** Immunofluorescence staining analysis of MitoTracker (red) and a Ca^2+^ indicator (Fluo-4 AM; green) in the vehicle, MGTMM, and MGTMM + FA. Scale bars = 20 μm. **g** The fluorescence intensity of (**f**) was measured in the vehicle, MGTMM, and MGTMM + FA. Values are means ± SDs. *n* = 3 for each group. ***p < 0.001 versus the MGTMM. **h** qRT-PCR showing the mRNA expression of fatty acid oxidation markers (*CPT1B*, *CD36*, and *PPARA*) in the vehicle, MGTMM, and MGTMM + FA. Values are means ± SDs. *n* = 6. *p < 0.05, ****p < 0.0001 versus the MGTMM. **i** Western blotting of a fatty acid oxidation marker (CPT1B) in the vehicle, MGTMM, and MGTMM + FA. GAPDH was used as the loading control.

We investigated whether FA improves cardiac function upon direct human cardiac reprogramming. To assess cardiac function, we analyzed the expression levels of ion channels by qRT-PCR (Fig. 5a, b). The expression of Ca^2+^ channel markers (*CACNA1C* and *RYR2*) was greater in the MGTMM + FA compared to the MGTMM (Fig. 5a). However, the levels of potassium channel markers (*KCNH2* and *KCNJ2*) were not significantly different between the two groups (Fig. 5b). Within CMs, mitochondria play a crucial role by producing over 90% of the ATP required for cardiac function^39^. Consequently, we investigated the expression levels of mitochondrial biogenesis markers (*TFAM*, *PPARGC1A*, and *SIRT1*) by qRT-PCR (Fig. 5c). *TFAM*, *PPARGC1A*, and *SIRT1* were upregulated in the MGTMM + FA compared with the MGTMM. Compared with those in the vehicle group, the protein levels of TFAM and PPARGC1A were elevated in the MGTMM group and further elevated in the MGTMM + FA group (Fig. 5d). To quantify the mitochondrial content in each group, we analyzed the ratio of mitochondrial DNA (mtDNA) to nuclear DNA (nDNA) by qRT-PCR. We found a significant increase in the mtDNA/nDNA ratio in the MGTMM + FA compared with the MGTMM (Fig. 5e).

Ca^2+^ is crucial for CM function, and the dynamics of mitochondrial Ca^2+^ cycling and storage during excitation‒contraction coupling (ECC) are strongly linked to cardiac physiology and pathophysiology^40–42^. Mitochondrial Ca^2+^ uptake is crucial for regulating cellular metabolism and provides the energy necessary for contraction^43–46^. To analyze the relationship between mitochondria and Ca^2+^ handling, we investigated MitoTracker and Fluo-4 AM by immunofluorescence staining (Fig. 5f, g). These colocalized signals were greater in the MGTMM + FA than in the MGTMM.

Fatty acid metabolism, oxidative phosphorylation, and mitochondrial biogenesis are characteristic functions of mature CMs. FA treatment increased the mRNA levels of *CPT1B*, *CD36*, and *PPARA* and the protein level of CPT1B (Fig. 5h, i). These findings indicate that FA enhanced cardiac function by improving ion channel function, mitochondrial biogenesis, and metabolism during cardiac reprogramming.

### Transcriptome analysis reveals the impact of MGTMM + FA on the cardiac reprogramming pathway

**Fig. 6:**
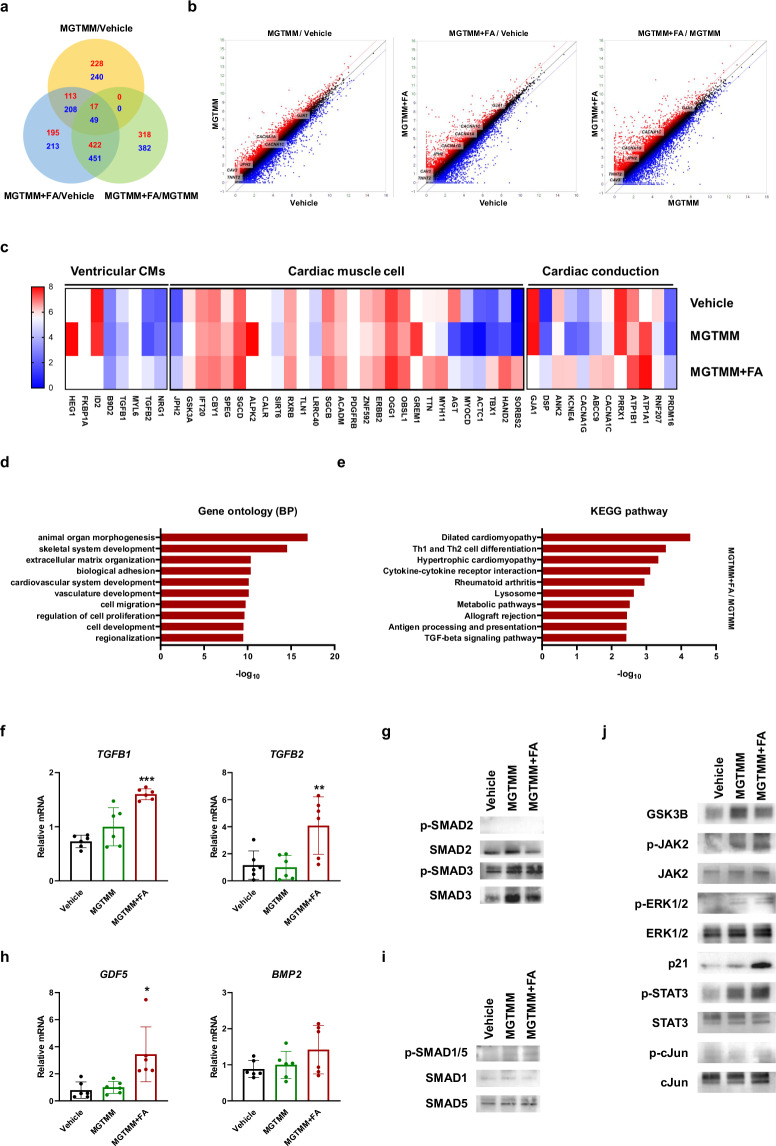
MGTMM + FA activates JAK2–STAT3 signaling and induces direct cardiac reprogramming. **a** Venn diagrams of upregulated and downregulated genes (with >2-fold change) between the vehicle, MGTMM, and MGTMM + FA. Upregulated (red); downregulated (blue). **b** Scatter plots showing transcript expression in the MGTMM compared with the vehicle (left), the MGTMM + FA compared with the vehicle (middle), and the MGTMM + FA compared with the MGTMM (right). **c** Clustered heatmap of DEGs, including cardiac conduction-, cardiac muscle cell-, and ventricular CM-related genes, in the vehicle, MGTMM, and MGTMM + FA. **d** GO analysis of upregulated genes in the MGTMM + FA compared with the MGTMM. **e** Pathway analysis of the 15 major enriched KEGG pathways in the MGTMM + FA group compared with those in the MGTMM group. qRT-PCR showing the mRNA expression of **f** TGFBs (*TGFB1* and *TGFB2*) and **h** GDFs (*GDF5* and *BMP2*) in the vehicle, MGTMM, and MGTMM + FA. Values represent the means ± SDs. *n* = 6 for each group. *p < 0.05, **p < 0.01, ***p < 0.001 versus the MGTMM. Western blotting of **g** SMADs p-SMAD2, SMAD2, p-SMAD3, SMAD3, **i** p-SMAD1/5, and SMAD1, and **j** JAK–STAT signaling pathway components (GSK3B, p-JAK2, JAK2, p-ERK1/2, ERK1/2, p21, p-STAT3, STAT3, p-cJun, and cJun) in the vehicle, MGTMM, and MGTMM + FA. GAPDH served as a loading control.

To understand the genes and signaling pathways involved in maturation resulting from co-treatment with FA, we analyzed the transcriptomes of the vehicle, MGTMM, and MGTMM + FA groups. Among the 25,737 genes, 358 genes in the MGTMM compared with the vehicle, 747 genes in the MGTMM + FA compared with the vehicle, and 757 genes in the MGTMM + FA compared with the MGTMM were upregulated (fold change > 2; Fig. 6a). In contrast, 497 genes in the MGTMM compared with the vehicle, 921 genes in the MGTMM + FA compared with the vehicle, and 882 genes in the MGTMM + FA compared with the MGTMM were downregulated (fold change > 2). Scatter plots highlighting upregulated cardiac-specific genes (*TNNT2*, *CAV3*, *JPH2*, *CACNA1A*, *CACNA1C*, and *GJA1*) are shown in Fig. 6b. Heatmap analysis further revealed upregulation of cardiac conduction, cardiac muscle cells, and ventricular CM-related genes in the MGTMM + FA compared with those in the MGTMM (Fig. 6c). To elucidate the biological processes regulated by differentially expressed genes (DEGs), we performed gene ontology (GO; Fig. 6d) and analyzed Kyoto Encyclopedia of Genes and Genomes (KEGG) pathway databases to identify active signaling pathways involved in the reprogramming processes (Fig. 6e). The most significantly regulated GO biological processes and KEGG pathways associated with these processes are shown in Supplementary Tables 1 and 2.

### RNA-seq analysis reveals the potential of JAK–STAT3 signaling in iCM maturation

To explore the common signaling pathways involved in reprogramming induced by MGTMM and FA treatment, we performed KEGG pathway analysis and examined the genes involved (Supplementary Tables 1 and 2). Compared with the vehicle, MGTMM upregulated the MAPK, PI3K-AKT, VEGF, Ca^2+^, and p53 signaling pathways (Supplementary Fig. 8a). Furthermore, MGTMM + FA upregulated the JAK–STAT and TGFB signaling pathways.

To validate these findings, we investigated ECM-receptor interactions and focal adhesions (Supplementary Fig. 8b–d). COL1A1 and laminin in the ECM and ITGAV, ITGA7, and ITGB3 in integrins were increased in the MGTMM and MGTMM + FA compared with the vehicle (Supplementary Fig. 8b, c). However, FN1 was lower in the MGTMM and MGTMM + FA compared with vehicle. ROCK1, ROCK2, TLN1, and DES in focal adhesions were highly expressed in the MGTMM and MGTMM + FA compared with the vehicle, whereas VCL and TLN2 were not significantly different among the vehicle, MGTMM, and MGTMM + FA groups (Supplementary Fig. 8d). These results suggest that MGTMM induced cardiac reprogramming by activating VEGF, ECM proteins, integrins, focal adhesions, and Ca^2+^ signaling.

To examine signaling pathways related to the maturation of iCMs following FA treatment, we analyzed the JAK–STAT and TGFB signaling pathways (Fig. 6f–j and Supplementary Fig. 9). TGFB1, TGFB2, and GDF5 were elevated in the MGTMM + FA compared with the MGTMM (Fig. 6f, h). The expression levels of BMP2 and BMP4 were not significantly different between the groups. Therefore, we further analyzed the downstream signaling pathways of TGFB1/2, SMAD2, and SMAD3 and the downstream signaling pathways of GDF5 and SMAD1/5 (Fig. 6g, i). The results revealed an increase in p-SMAD3 in the MGTMM + FA compared with the MGTMM, whereas p-SMAD1/5 was not significantly different. Additionally, p-JAK2, p-STAT3, and p21 levels in the JAK–STAT3 pathway were increased in the MGTMM + FA compared with the MGTMM, whereas the p-cJun level was not significantly different between the groups (Fig. 6j). These findings indicate that FA contributed to the maturation of iCMs by influencing the JAK–STAT and TGFB signaling cascades.

### The JAK2–STAT3 signaling pathway modulates iCM maturation upon treatment with FA

**Fig. 7:**
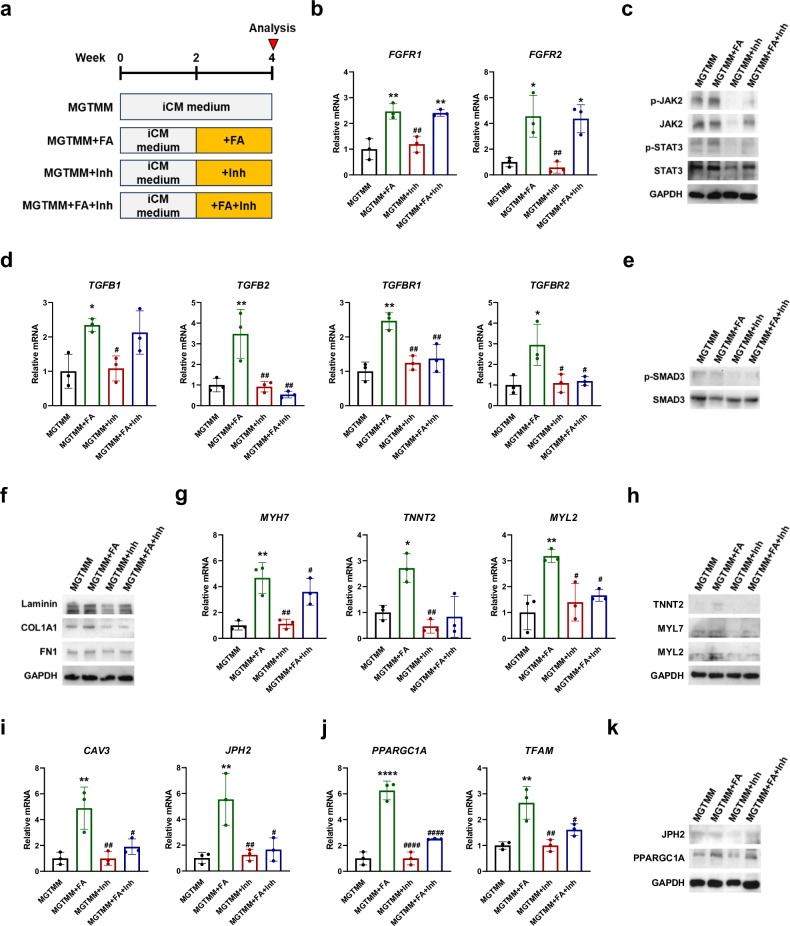
Inhibition of the JAK2–STAT3 signaling pathway affects iCM maturation. **a** Schematic diagram showing JAK2–STAT3 knockdown in the MGTMM and MGTMM + FA. **b** qRT-PCR showing the mRNA expression of *FGFR1* and *FGFR2* in the MGTMM, MGTMM + FA, MGTMM + Inh, and MGTMM + FA + Inh. The values are the means ± SDs. *n* = 3 for each group. *p < 0.05, **p < 0.01 versus the MGTMM and ^#^*p* < 0.05, ^##^*p* < 0.01 versus the MGTMM + FA. **c** Western blotting of JAK2–STAT3 signaling (p-JAK2, JAK2, p-STAT3, and STAT3) in the MGTMM, MGTMM + FA, MGTMM + Inh, and MGTMM + FA + Inh. GAPDH served as a loading control. **d** qRT-PCR showing the mRNA expression of genes involved in the TGFB signaling pathway (*TGFB1*, *TGFB2*, *TGFBR1*, and *TGFB2*) in the MGTMM, MGTMM + FA, MGTMM + Inh, and MGTMM + FA + Inh groups. The values are the means ± SDs. *n* = 3 for each group. *p < 0.05, **p < 0.01 versus the MGTMM and ^#^*p* < 0.05, ^##^*p* < 0.01 versus the MGTMM + FA. Western blot analysis of **e** p-SMAD3 and **f** ECM markers (laminin, COL1A1, and FN1) in the MGTMM, MGTMM + FA, MGTMM + Inh, and MGTMM + FA + Inh. **e** SMAD3 or **f** GAPDH served as a loading control. **g** qRT-PCR showing the mRNA expression of CM markers (*MYH7*, *TNNT2*, and *MYL2*) in the MGTMM, MGTMM + FA, MGTMM + Inh, and MGTMM + FA + Inh. The values are the means ± SDs. *n* = 3 for each group. *p < 0.05, **p < 0.01 versus the MGTMM and ^#^*p* < 0.05, ^##^*p* < 0.01 versus MGTMM + FA. **h** Western blotting of CM markers (TNNT2, MYL7, and MYL2) in the MGTMM, MGTMM + FA, MGTMM + Inh, and MGTMM + FA + Inh. GAPDH served as a loading control. qRT-PCR showing the mRNA expression of **i** T-tubule markers (*CAV3* and *JPH2*) and **j** mitochondrial biogenesis markers (*PPARGC1A* and *TFAM*) in the MGTMM, MGTMM + FA, MGTMM + Inh, and MGTMM + FA + Inh. The values are the means ± SDs. *n* = 3 for each group. **p < 0.01, ****p < 0.0001 versus the MGTMM and ^#^*p* < 0.05, ^##^*p* < 0.01, ^####^*p* < 0.0001 versus the MGTMM + FA. **k** Western blotting of mature CM markers (JPH2 and PPARGC1A) in the MGTMM, MGTMM + FA, MGTMM + Inh, and MGTMM + FA + Inh. GAPDH served as a loading control.

Additionally, owing to the remarkable activation of the JAK2–STAT3 signaling pathway in the MGTMM + FA, which exhibited both structural and functional maturation compared with the MGTMM, we further investigated the roles of the JAK2–STAT3 signaling pathway in iCM maturation. We treated MGTMM- and MGTMM + FA-treated cells with cryptotanshinone, a JAK2–STAT3 inhibitor, from week 2 to 4 and performed qRT-PCR and western blot analyses (Fig. 7a). The mRNA expression levels of *FGFR1* and *FGFR2* were significantly higher in the MGTMM + FA and MGTMM + FA + Inh than in the MGTMM and MGTMM + Inh (Fig. 7b). These results indicated that the FGF signaling pathway was activated in FA-treated iCMs. To analyze the effects of the inhibitor cryptotanshinone, we investigated the JAK2–STAT3 signaling pathway by western blot analysis (Fig. 7c). The protein levels of p-JAK2, JAK2, p-STAT3, and STAT3 were lower in the MGTMM + Inh and MGTMM + FA + Inh than in the MGTMM and MGTMM + FA. These results demonstrate that cryptotanshinone inhibited the JAK2–STAT3 signaling pathway.

The JAK2–STAT3 signaling pathway promotes the transcription of TGFB, collagen, and MYH7. Next, to investigate whether TGFB signaling was affected during iCM maturation, we analyzed markers of the TGFB signaling pathway by qRT-PCR (Fig. 7d). *TGFB1*, *TGFB2*, *TGFBR1*, and *TGFBR2* were significantly lower in the MGTMM + Inh than in the MGTMM + FA, and there was no significant difference in the MGTMM + Inh compared with the MGTMM. *TGFB2*, *TGFBR1*, and *TGFBR2* were decreased in the MGTMM + FA + Inh compared to the MGTMM + FA, but *TGFB1* was not significantly different between the MGTMM + FA and MGTMM + FA + Inh. We also observed increased phosphorylation of SMAD3 in the MGTMM + FA compared with the MGTMM and decreased phosphorylation in the MGTMM + Inh and MGTMM + FA + Inh compared with the MGTMM + FA (Fig. 7e). To examine the regulation of collagen by JAK2–STAT3 inhibition, we performed western blotting for ECM markers (laminin, COL1A1, and FN1) (Fig. 7f). Laminin and COL1A1 were lower in the MGTMM + FA + Inh than in the MGTMM + FA, whereas FN1 was not significantly different between the MGTMM + FA and MGTMM + FA + Inh. These findings demonstrate that during direct cardiac reprogramming, the JAK2–STAT3 signaling pathway regulated TGFB2, TGFBR1, TGFBR2, laminin, and COL1A1.

To examine whether the JAK2–STAT3 signaling pathway induces cardiac differentiation in FA-treated iCMs, we analyzed cardiac markers (*MYH7*, *TNNT2*, and *MYL2*) by qRT-PCR (Fig. 7g). The *MYH7*, *TNNT2*, and *MYL2* levels were lower in the MGTMM + Inh than in the MGTMM + FA. TNNT2 levels were not significantly different between the MGTMM + FA and MGTMM + FA + Inh, whereas *MYH7* and *MYL2* levels were lower. The protein levels of TNNT2, MYL7, and MYL2 were also lower in the MGTMM + Inh and MGTMM + FA + Inh than in the MGTMM + FA (Fig. 7h). To investigate the induction of cardiac maturation via JAK2–STAT3 signaling, we examined T-tubule markers (*CAV3* and *JPH2*) and mitochondrial biogenesis markers (*PPARGC1A* and *TFAM*) by qRT-PCR (Fig. 7i, j). The mRNA levels of *CAV3*, *JPH2*, *PPARGC1A*, and *TFAM* were significantly lower in the MGTMM + Inh and MGTMM + FA + Inh groups than in the MGTMM + FA group; the protein expression of JPH2 and PPARGC1A was also significantly different (Fig. 7k). Collectively, these results demonstrate that the JAK2–STAT3 signaling pathway contributed to the maturation of iCMs by regulating the TGFB (TGFB2, TGFBR1, and TGFBR2) signaling pathway.

## Discussion

Our findings suggest that the combination of FA effectively promoted the maturation and direct reprogramming of fibroblasts into CMs via the combination of three (in mice) or five (in humans) cardiac transcription factors: MGT in mice and MGTMM in humans. FA synergistically enhanced the expression of these factors, resulting in significant improvements in cardiac function. Specifically, during direct cardiac reprogramming, this combination facilitated critical processes, including the maturation of CMs, the formation of T-tubule structures, the regulation of Ca^2+^ exchange dynamics, and the induction of mitochondrial biogenesis. Moreover, the integration of small molecules has the potential to enhance reprogramming in conjunction with transcription factors, thereby playing a pivotal role in promoting CM maturation during cardiac differentiation^11^.

To compare our differentiation method with other direct cardiac reprogramming methods, including the MGT approach, we focused on both enhancing the conversion efficiency and inducing the transition to mature CMs through growth factor treatment. While other studies have used combinations of cytokines and the regulation of signaling pathways, we used only FA, which was highly efficient and improved the efficiency of the maturation of CMs. Additionally, we explored less understood reprogramming signaling pathways, providing new insights into the molecular mechanisms driven by FA.

However, the use of only FA may overlook other potential inducers, and our study lacks direct comparisons with other methods utilizing signaling regulation. Additionally, focusing on specific signaling pathways may have limited the exploration of other important pathways. Moreover, while our in vitro results are promising, a limitation of our study is that in vivo experiments are necessary for validating our findings and determining efficacy and safety.

An encouraging strategy for enhancing the efficiency, quality, and speed of direct reprogramming involves the simultaneous suppression of the TGFB and WNT signaling pathways^47^. Furthermore, targeting signaling pathways, such as the ROCK, NOTCH, C-C chemokine, p38 mitogen-activated protein kinase, and PI3K/AKT pathways, has been identified as a means of increasing reprogramming efficiency^24,26,47–50^. A noteworthy discovery was the effectiveness of FFV in overcoming obstacles encountered in later stages of cardiac reprogramming^26^. Our findings highlight that the combination of FA resulted in a significant population of iCMs and substantial induction of mature iCMs.

Transcriptional profiling data revealed the upregulation of pathways such as the MAPK, PI3K-AKT, VEGF, Ca^2+^, and NF-κB signaling pathways, ECM-receptor interaction, focal adhesion, HIF1 signaling, and p53 signaling in the MGTMM group compared with those in the vehicle group. Furthermore, our results demonstrate that MGTMM induced the transcription of cardiac genes, ECM components, integrins, focal adhesion proteins, and Ca^2+^ channels during the direct cardiac reprogramming of human fibroblasts. The ECM interacts with integrins, initiating downstream signaling pathways such as focal adhesion and actin-cytoskeletal regeneration. Our results revealed increased ROCK1, ROCK2, TLN1, and DES levels in the MGTMM compared with those in the vehicle. These results indicate that MGTMM promoted the transcription of cardiac genes, ECM components, and Ca^2+^ channels, followed by the activation of focal adhesion and actin cytoskeleton markers during the transdifferentiation of fibroblasts into CMs.

Our research further underscores the pivotal role of the JAK2–STAT3 signaling pathway in cardiac maturation. The JAK2–STAT3 pathway, which is activated by FGF signaling, is critical in various physiological processes, including immune responses, cell division, cell death, and tumorigenesis^51^. Within the heart, the JAK2–STAT3 pathway is essential for maintaining cardiac homeostasis and responds to various cytokines, such as IL6, granulocyte colony-stimulating factor, leptin, and erythropoietin^52^. Transcriptional profiling data revealed the significant upregulation of genes associated with the JAK2–STAT3 signaling pathway in the MGTMM + FA group compared with the MGTMM group. On the basis of this observation, we conclude that the JAK2–STAT3 signaling pathway was crucial for the maturation of CMs activated by the combination of FA during direct reprogramming.

In cardiac hypertrophy and the fibrotic response, the JAK2–STAT3 signaling pathway activates TGFB, COL1A1, and MYH7 transcription, contributing to cardiac remodeling and dysfunction^53^. Our results revealed no significant differences in the TGFB signaling pathways, such as TGFB1, TGFBR1, and TGFBR2, between the MGTMM + FA and MGTMM + FA + Inh. However, TGFB2, TGFBR2, and TGFB2-downstream signaling (p-SMAD3) were reduced in the MGTMM + FA + Inh compared with the MGTMM + FA. These findings suggest that TGFB2–TGFBR2–p-SMAD3 activation occurred via the JAK2–STAT3 signaling pathway during FA-mediated direct cardiac reprogramming. During heart development, TGFB plays critical roles in populating the embryonic heart with CMs^54^. The TGFB signaling pathway triggers the expression of cardiogenic markers in fibroblasts and promotes the maturation of CMs^55^. In the present study, cardiac genes, T-tubule markers, and mitochondrial biogenesis markers were downregulated in the MGTMM + FA + Inh group compared with those in the MGTMM + FA group. These findings demonstrate that the TGFB2–TGFBR2 signaling pathway promoted CM maturation through FA via the JAK2–STAT3 signaling pathway. Thus, generating iCMs through the use of FA could be pivotal in treating CVDs.

## Supplementary information

Supplementary Information
