# Supplementary material for: FGF4 and ascorbic acid enhance the maturation of induced cardiomyocytes by activating JAK2–STAT3 signaling
Source: Exp Mol Med. 2024 Oct 1;56(10):2231–45. doi: 10.1038/s12276-024-01321-z (PMC11541553; doi:10.1038/s12276-024-01321-z)
Supplement: Supplementary file 1 — Supplementary Information [file 12276_2024_1321_MOESM1_ESM.pdf]

Supplementary data to Jun et al: FGF4 and Ascorbic acid enhance the maturation of induced cardiomyocytes by activating JAK2-STAT3 signaling

Corresponding author:

Do-Sun Lim.

Department of Cardiology, Cardiovascular Center, College of Medicine, Korea University

145 Anam-ro, Seongbuk-gu, Seoul, 02841, Republic of Korea

E-mail address: dslmd@naver.com (D.S. Lim)

Contents:

1. SUPPLEMENTARY MATERIALS AND METHODS
2. SUPPLEMENTARY FIGURES 1 to 9
3. SUPPLEMENTARY TABLES 1 to 4

1. SUPPLEMENTARY MATERIALS AND METHODS

Mouse fibroblast isolation and culture

For mouse embryonic fibroblast (MEF) isolation, embryos harvested from 13.5-day pregnant mice were washed with PBS and inverted 2-3 times to remove blood. The head and red organs were carefully dissected and transferred to a new Petri dish in 0.25% trypsin-EDTA solution, and the fetal tissue was thoroughly minced with scissors. The tissue fragment was transferred into a falcon tube and incubated at 37 °C for 15 min. After 5 min of incubation, all tissue fragment suspensions were gently pipetted up and down to dissociate the cells. After incubation, MEFs were filtered using a 40 μm cell strainer and centrifuged. Harvested MEFs were passaged in DMEM/High glucose (Hyclone Laboratories Inc., South Logan, UT, USA) containing 10% FBS (Hyclone Laboratories Inc.) and 1% penicillin/streptomycin (P/S; Gibco, Life Technologies, USA) at 37 °C on 0.1% gelatin-coated 100 mm culture dishes. Mouse cardiac fibroblasts (MCFs) were harvested from wild-type C57BL/6 mice with PBS pumped into heart to remove blood. Heart tissue was minced into small pieces using scissors and transferred into Falcon tubes. Heart sections were incubated with 0.2% collagenase type II at 37 °C for 20 min. After 5 min of incubation, the tissue was gently pipetted several times to allow for tissue dissociation. Digested tissue was filtered through a 40 μm cell strainer followed by centrifugation. MCFs were incubated on 0.1% gelatin-coated 100 mm culture dish in IMDM medium containing 20% FBS and 1% P/S at 37 °C.

Mouse fibroblasts at early passage numbers (P3-P4) were used in all the experiments.

Human fibroblast culture

NHDFs were purchased from Lonza Inc. (CC2509, Switzerland). NHDFs were cultured in fibroblast growth medium (FGM2 BulletKit; CC-3132, Lonza) and grown to 70-80% confluence. NHDFs were passaged at a ratio of 1:4 using trypsin/EDTA (T/E; Thermo Fisher Scientific, Cleveland, OH, USA) for dissociation into a single-cell suspension. The medium was changed every 2 days.

For all experiments, NHDFs at early passage number (P6) were used.

Virus Packaging

HEK 293GPG packaging cells were maintained in growth media containing DMEM/low glucose, 10% FBS, 1% P/S, 1 μg/mL tetracyclin (Sigma-Aldrich, St Louis, MO, USA), 2 μg/mL puromycin (P8833, Sigma-Aldrich), and 300 μg/mL G418 (ant-gn-1, Invivogen,

USA). One day before transfection, 1.65 x 10^6 cells were seeded per well in 6 well plates with growth media without antibiotics. The next day, retroviral vectors were introduced into 293GPG cells using Lipofectamine 3000 (L3000-015, Invitrogen, Carlsbad, CA, USA) transfection reagent according to the manufacturer's recommendations. The retroviral vectors pMXs-puro-GFP (plasmid #74203) and pMx-puro-MGT (plasmid #111809) were purchased from Addgene (USA). Retroviral supernatant was collected at 24, 48, and 72 h post-transfection and filtered through a 0.45-μm filter. Viruses were frozen at -80 °C for future use.

For human cardiac reprogramming, pLVX-EF1α-MGT, pLVX-EF1α-MESP1-puro, and pLVX-EF1α-MYOCD-puro were constructed in the laboratory and used as packaging plasmid. Lentiviral plasmids were transfected into 293T cells using Lipofectamine 3000 (Invitrogen). The lentivirus particles were collected 72 h after transfection.

Mouse direct cardiac reprogramming

Before viral transduction, MEFs were seeded onto Matrigel (BD Biosciences, Franklin Lakes, NJ, USA)-coated plate at 4.5 x 10^4 cells per well in a 6-well plate or 2 × 10^4 cells per 12-well plate in the same medium but without antibiotics for 24 h. Approximately 1 × 10^4 MCFs were plated in a matrigel-coated 6-well plate. MEFs and MCFs were infected with the retroviral supernatant for 24 h, washed with PBS, and switched to cardiac reprogramming medium (iCM medium) composed of DMEM/low glucose: Medium 199 (4:1, Hyclone, Gibco), 10% FBS, and 1% P/S. The induction medium was changed every 2 days. For positive selection, puromycin at 2 μg/mL was added to MEFs 3 days after viral infection and was maintained in iCM medium at the concentration of 1 μg/mL. After 2 weeks of direct reprogramming, the medium was changed to StemPro-34 SF medium (GIBCO, 10639-011), GlutaMAX (10 μl/ml, GIBCO, 35050-061) and 1% P/S (maturation factor medium) with/without maturation factors. Maturation factor medium was changed every 2-3 days. For screening of maturation factors, various growth factors used FGF2 10ng/ml, FGF10 50ng/ml, FGF4 10ng/ml, Ascorbic Acid (AA) 200μg/ml, VEGF 5ng/ml, TGFβ1 1ng/ml, and 1-Thioglycerol (1-TG) 450μM, either alone or in combination in stempro-34 SF medium.

Human direct cardiac reprogramming

One day before reprogramming, NHDFs were seeded into gelatin-coated 6-well plates at a density of 4 × 10^4 cells/well. For human direct cardiac reprogramming, pLVX lentiviruses

encoding MGT, MESP1, and MYOCD infected the NHDFs in the iCM medium without antibiotics. On day 2, the medium was changed to the iCM medium for 2 weeks. The iCM was changed every two days. At week 2, the culture medium was replaced with RPMI-1640 medium supplemented with 2% B27 and 10% FBS. Cells were treated with 10 ng/mL FGF4+200 μg/mL Ascorbic acid from week 2 to week 4. The RPMI-1640 medium was changed every two or three days.

To inhibit the JAK2-STAT3 signaling pathway, cryptotanshinone at 10 μM was added to MGTMM-treated NHDFs with/without FA from week 2 to week 4, and the medium was changed every two or three days.

qRT-PCR

Total RNA was extracted from each group using the TRIzol reagent (TR-118; MRC Inc., Cincinnati, OH, USA) according to the manufacturer's instructions. After adding chloroform (C2432, Sigma-Aldrich), the RNA-containing aqueous phase was transferred to a new tube, and isopropanol (2-propanol, 190764, Sigma-Aldrich) was used for RNA precipitation. Subsequently, RNA was rinsed with 75% ethanol and air-dried. RNA concentration and purity were measured using a Nanodrop spectrophotometer (ND-1000; Thermo Fisher Scientific). The mRNA was reverse-transcribed into cDNA using M-MLV reverse transcriptase (28025-013, Invitrogen, Carlsbad, CA, USA) at 37 °C for 50 min in a 20 μL reaction. qRT-PCR was performed using the iQTM SYBR Green supermix (170-8880, Bio-Rad Laboratories, Hercules, CA, USA), and the results were recorded using the MYiQ2 Detection System (Bio-Rad Laboratories). Relative mRNA expression levels were calculated and normalized to glyceraldehyde 3-phosphate dehydrogenase (GAPDH) as the reference gene. The primer sequences used for qRT-PCR are listed in Supplementary Table 3.

Transient Ca2+ analysis

For transient Ca2+ analysis, directly reprogrammed cells were re-seeded in a confocal dish (213350; SPL, Seoul, Korea) at a density of 1.5 x 10^4 cells per dish. Ca2+ imaging was performed according to the manufacturer's instructions. Briefly, cells were loaded for 30 min with 5 μM Fluo-4 AM (F14201, Invitrogen) in HBSS medium at 37 °C. Nuclei were stained with 1 μg/mL of Hoechst 33342. Following the staining process, iCMs were washed with PBS and incubated in fresh medium for an additional 30 min to de-esterify the fluorescence.

Ca2+ imaging was performed using a confocal fluorescence microscope (LSM800, Carl Zeiss).

MitoTracker

Active mitochondria in iCMs were labeled using a standard experimental protocol with MitoTracker (Red CM-H_2XRos, M7513, Thermo Fisher Scientific). Concisely, iCMs at week 4 were re-seeded onto a confocal dish (213350, SPL) with a density of 1.5×10^4 cells per dish and then incubated with 300nM MitoTracker probe in DMSO for 30 min at 37 °C. Nuclei were stained with 1 μg/mL of Hoechst 33342. After staining live cells with MitoTracker, the iCMs were washed with PBS and replaced with fresh, pre-warmed media. The cells were analyzed using a confocal fluorescence microscope (LSM800, Carl Zeiss).

2. SUPPPLEMENTARY FIGURES

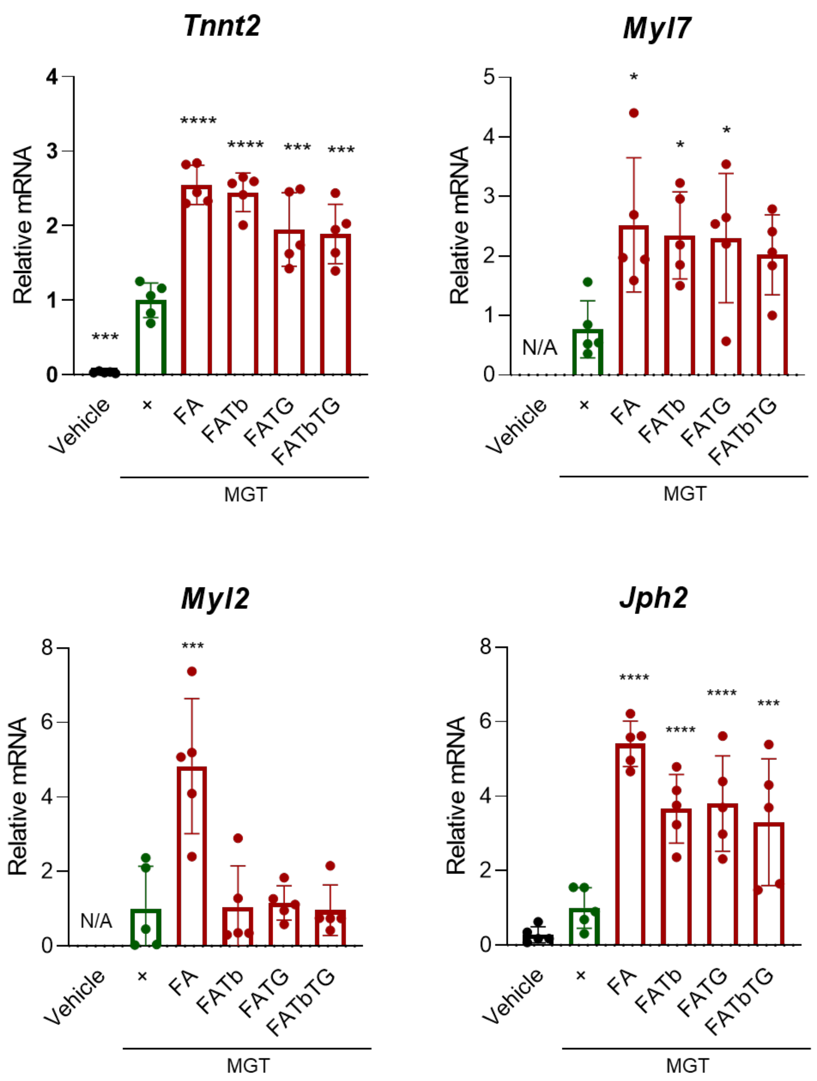

Supplementary Fig. 1. FA with other factors enhanced the expression of cardiac-related markers

mRNA expression of *Tnnt2*, *Myl7*, *Myl2*, and *Jph2* in MGT transfected cells, treated with FA, or FA in combinations with two other factors (Tgfb1 and 1-TG). The mRNA expression was shown using qRT-PCR (n = 5). All data are presented as mean ± SD. *p<0.05, **p<0.01, ***p<0.001, and ****p<0.0001 versus the MGT group. FA, FGF4 and ascorbic acid; 1-TG, 1-thioglycerol; Tgfb1, transforming growth factor b1.

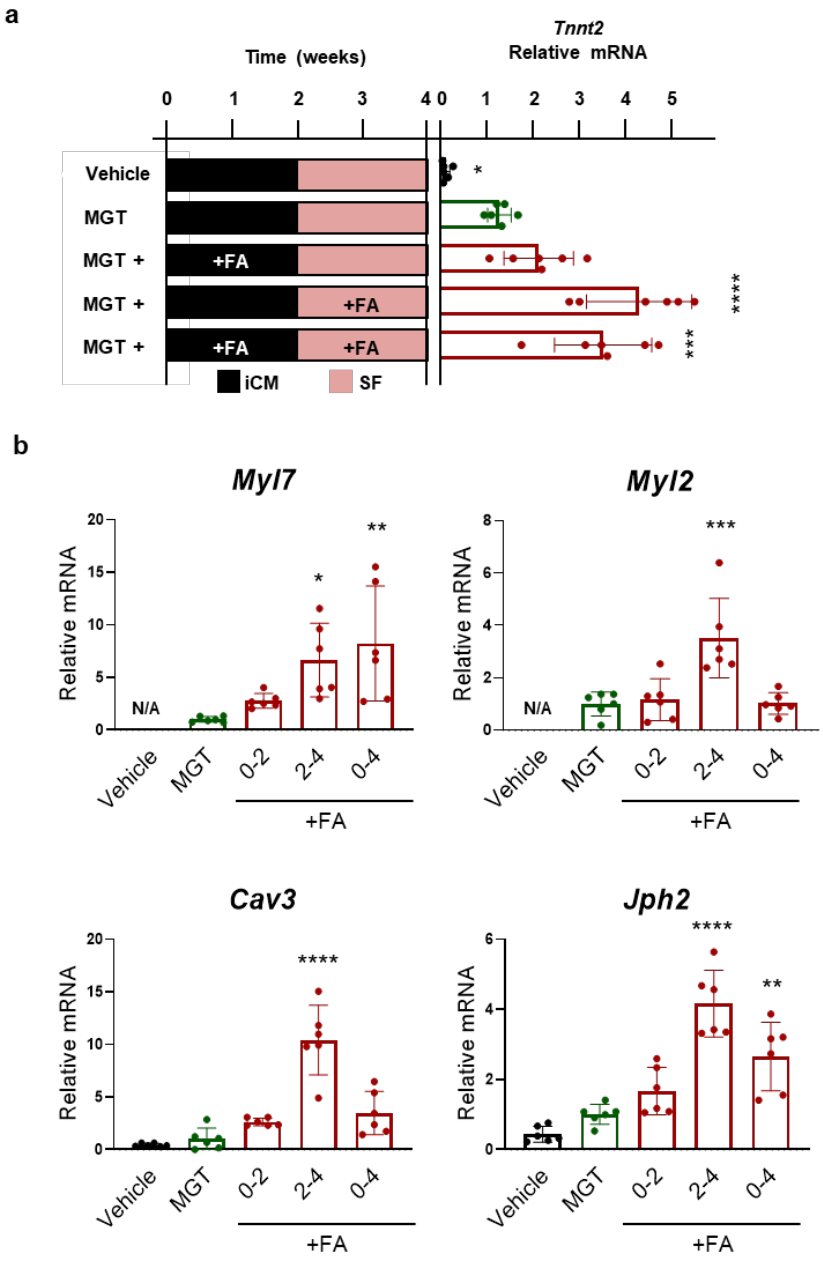

Supplementary Fig. 2. The timing of FA treatment regulates the conversion into iCMs
(a) Schematic representation of the strategy to determine the optimal timing of FA treatment for iCM maturation. The mRNA expression of *Tnnt2* for each condition is shown using qRT-PCR (right, n = 6). **(b)** qRT-PCR for *Myl7*, *Myl2*, *Cav3* and *Jph2* expression in iCM derived from MEFs (n = 6). All data are presented as mean ± SD. *p<0.05, **p<0.01, ***p<0.001, and ****p<0.0001 versus the MGT. FA, FGF4 and ascorbic acid; iCM, induced cardiomyocytes; MEF, mouse embryonic fibroblasts.

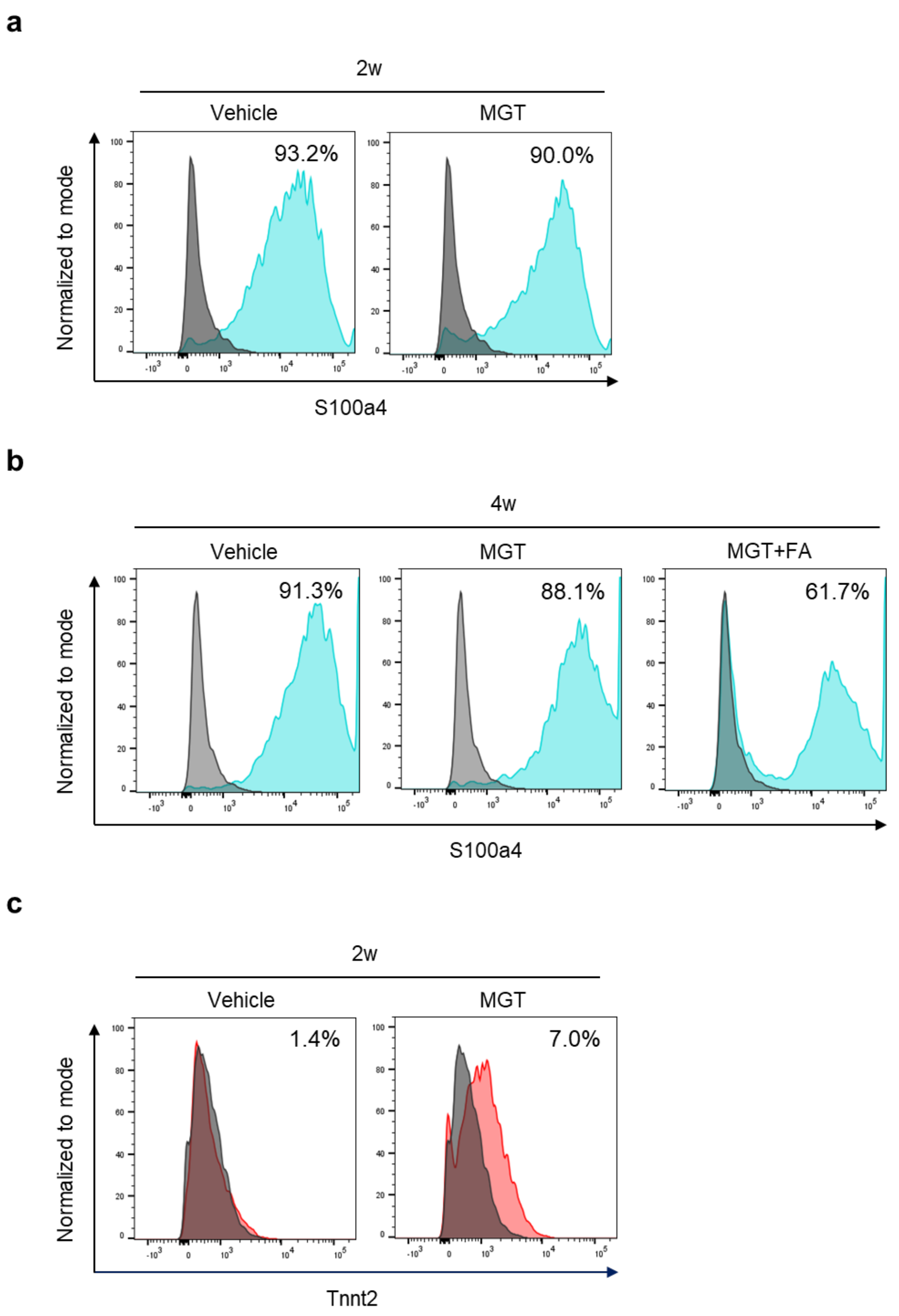

Supplementary Fig. 3. Proportion of cardiomyocytes and fibroblasts at week 2 and week 4

(a) Flow cytometry analysis showed the expression of fibroblasts at 2 weeks in vehicle and MGT. **(b)** The percentage of fibroblast cells at 4 weeks with vehicle, MGT, and MGT+FA. **(c)** Cardiomyocyte-positive cells were demonstrated at 4 week in vehicle and MGT.

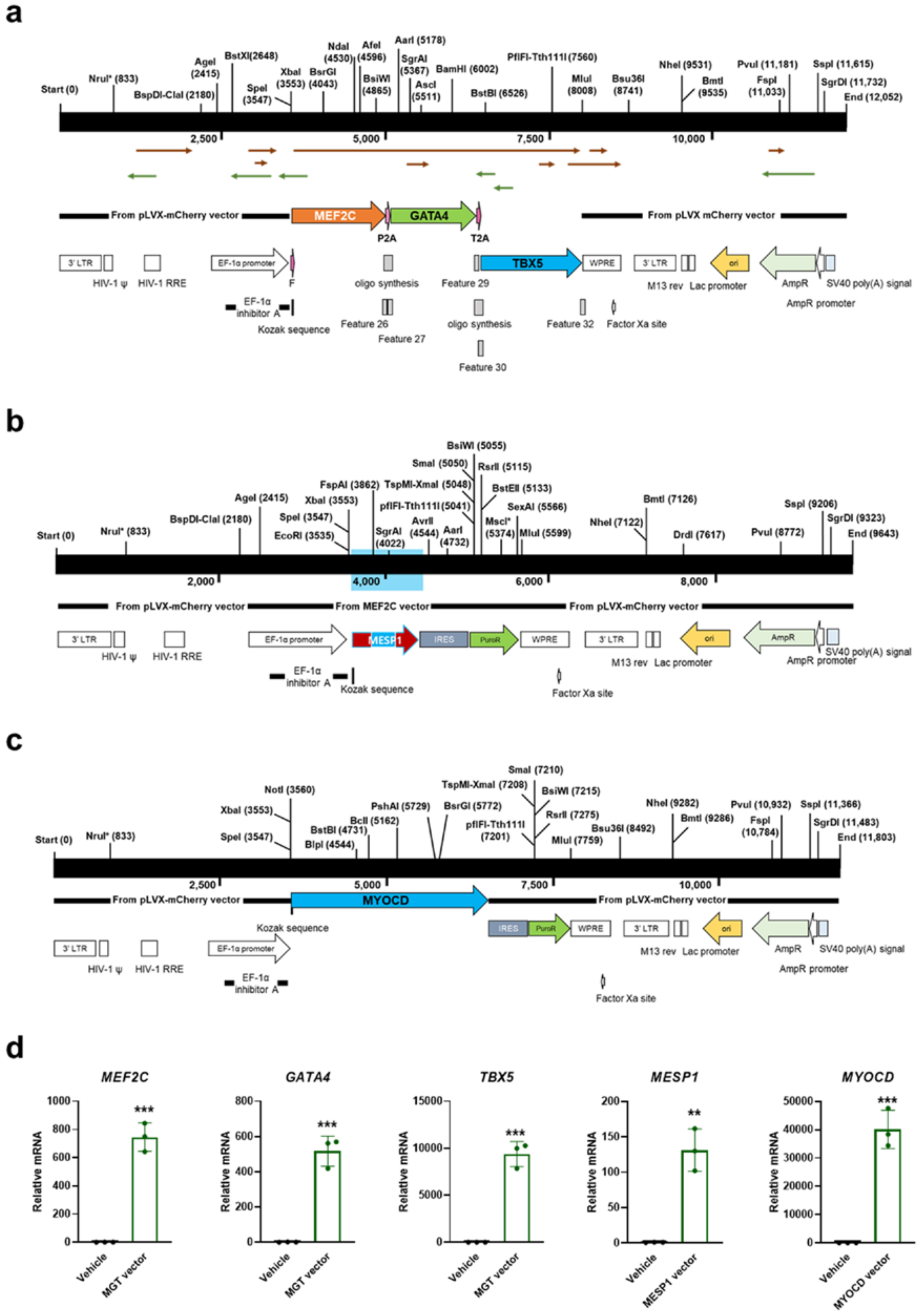

Supplementary Fig. 4. Transfection with lentiviral vectors resulted in the overexpression of target genes

Schematic diagram showing the lentiviral vectors **(a)** MGT, **(b)** MESP1, and **(c)** MYOCD. **(d)** qRT-PCR of transcription factors *MEF2C*, *GATA4*, and *TBX5* in vehicle and MGT vectors, *MESP1* in vehicle and MESP1 vectors, and *MYOCD* in vehicle and MYOCD vectors. Values are means ± SD. n=3 for each group. $^{**}p < 0.01$, $^{***}p < 0.001$ versus vehicle.

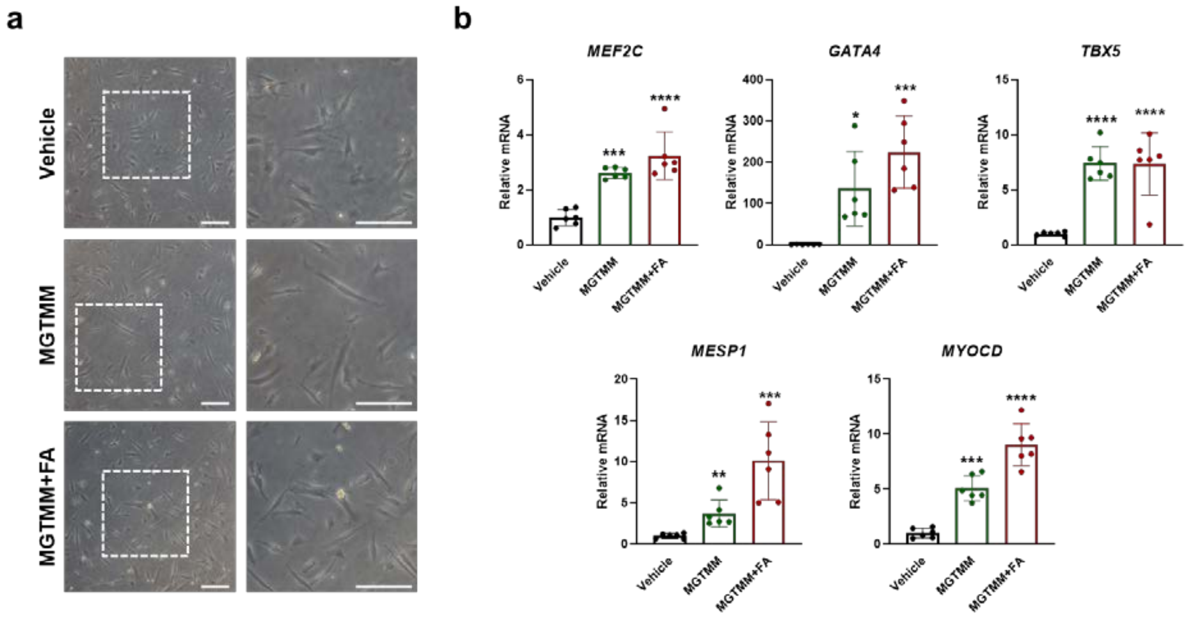

Supplementary Fig. 5. Lentiviral infection was employed to induce transdifferentiation into iCMs

(a) Bright-field images of the vehicle, MGTMM, and MGTMM+FA at week 4. Scale bars = 100 μm. **(b)** qRT-PCR of transcription factors (*MEF2C*, *GATA4*, *TBX5*, *MESP1*, and *MYOCD*) in vehicle, MGTMM, and MGTMM+FA. Values are means ± SD. n=4 for each group. *p < 0.05, **p < 0.01, ***p < 0.001, ****p < 0.0001 versus vehicle.

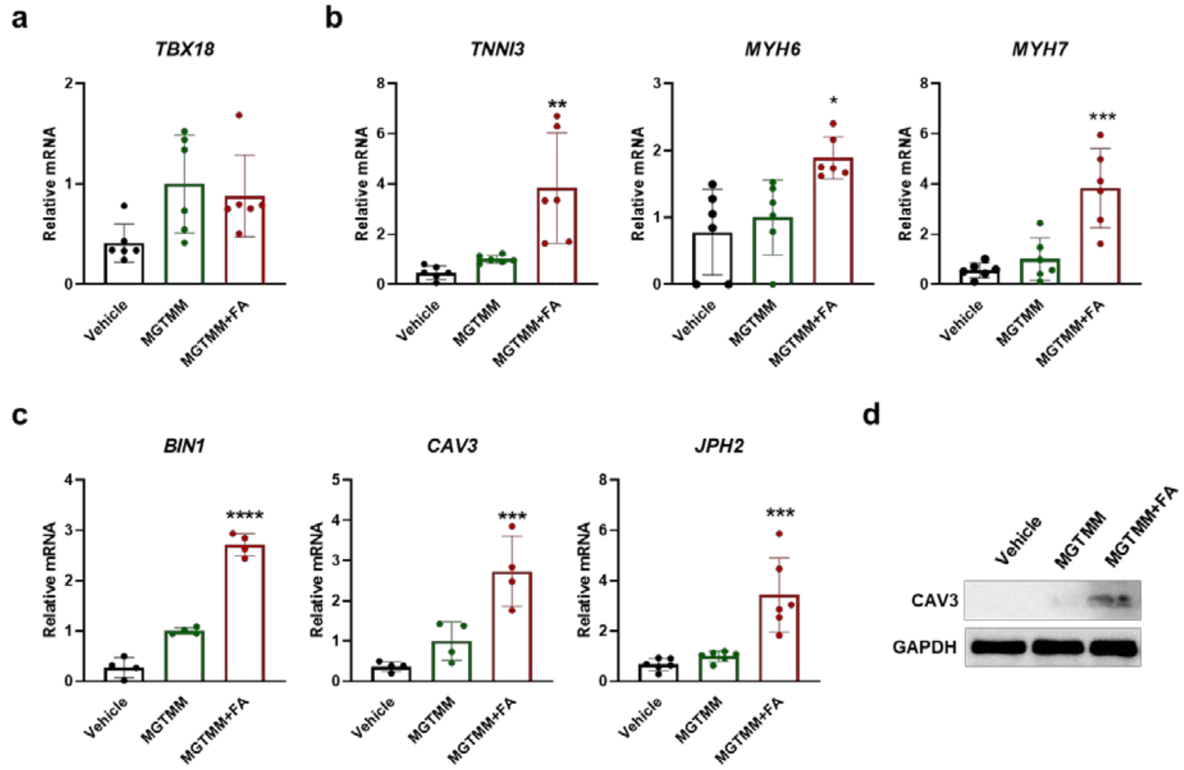

Supplementary Fig. 6. FA promotes cardiac differentiation

qRT-PCR showing the mRNA expression of **(a)** a nodal CM marker (*TBX18*), **(b)** CM markers (*TNNI3*, *MYH6*, and *MYH7*), and **(c)** T-tubule markers (*BIN1*, *CAV3*, and *JPH2*) in the vehicle, MGTMM, and MGTMM+FA groups. Values represent means ± SD. n = 6 for each group. *p < 0.05, **p < 0.01, ***p < 0.001, ****p < 0.0001 versus MGTMM. **(d)** Western blot analysis of the t-tubule marker CAV3 in the vehicle, MGTMM, and MGTMM + FA groups. GAPDH was used as the loading control.

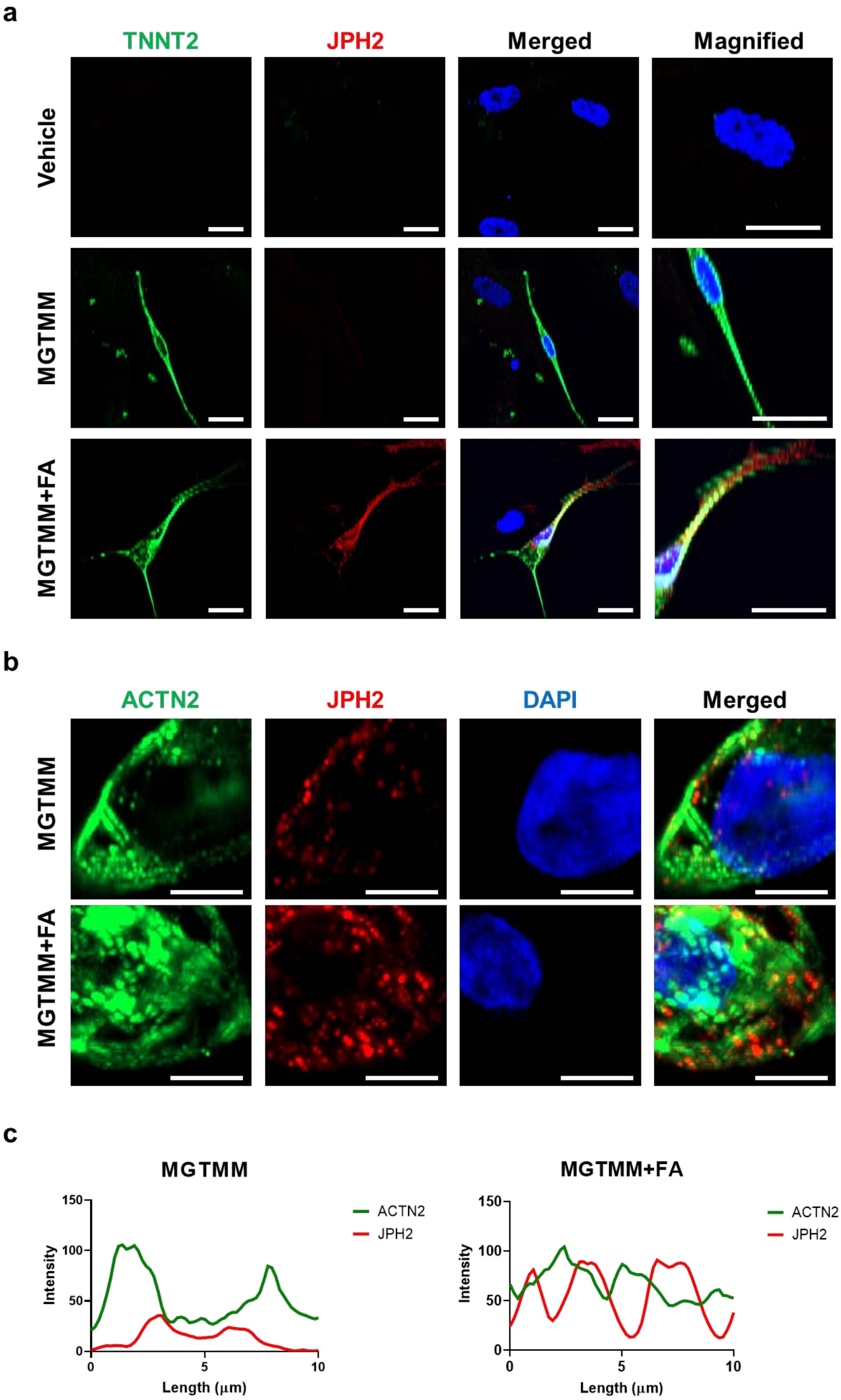

a
TNNT2
JPH2
Merged
Magnified
Vehicle
MGTMM
MGTMM+FA
b
ACTN2
JPH2
DAPI
Merged
MGTMM
MGTMM+FA
c
MGTMM
MGTMM+FA
Intensity
Length (μm)
ACTN2
JPH2
150
100
50
0
5
10

Supplementary Fig. 7. FA promotes cross structure of sarcomeres and t-tubules

Immunofluorescence analysis of **(a)** total CM marker (TNNT2; green) and t-tubule marker (JPH2; red), and **(b)** sarcomere marker (ACTN2; green) and t-tubule marker (JPH2; red) in the vehicle, MGTMM, and MGTMM+FA groups. Scale bars = 20 μm **(a)** and 5 μm **(b)**. The nuclei were stained with DAPI (blue). **(c)** Signal intensity plots showing positions of ACTN2 (green) and JPH2 (red).

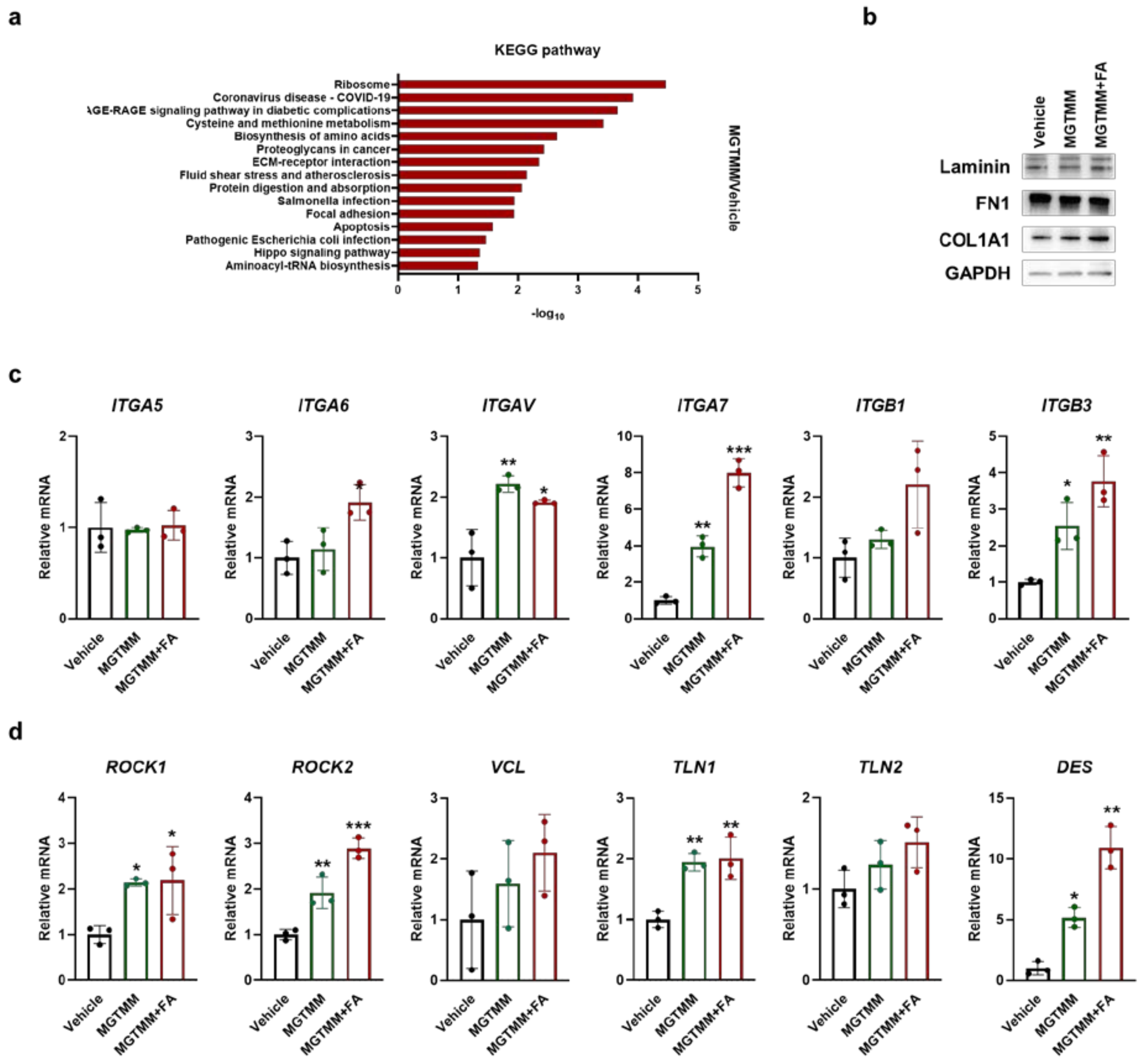

Supplementary Fig. 8. MGTMM transfection increases ECMs-Integrins-Focal adhesions

(a) Pathway analysis of the major 15 enriched KEGG pathways in MGTMM compared to the vehicle. **(b)** Western blotting of ECM markers (Laminin, COL1A1, and FN1) in the vehicle, MGTMM, and MGTMM+FA groups. GAPDH served as a loading control. qRT-PCR exhibiting mRNA expression of **(c)** integrins (*ITGA5*, *ITGA6*, *ITGAV*, *ITGA7*, *ITGB1*, and *ITGB3*) and **(d)** focal adhesions (*ROCK1*, *ROCK2*, *VCL*, *TLN1*, *TLN2*, and *DES*) in the vehicle, MGTMM, and MGTMM+FA groups. Values are means ± SD. n = 3 for each group. *p < 0.05, **p < 0.01, ***p < 0.001 versus the vehicle.

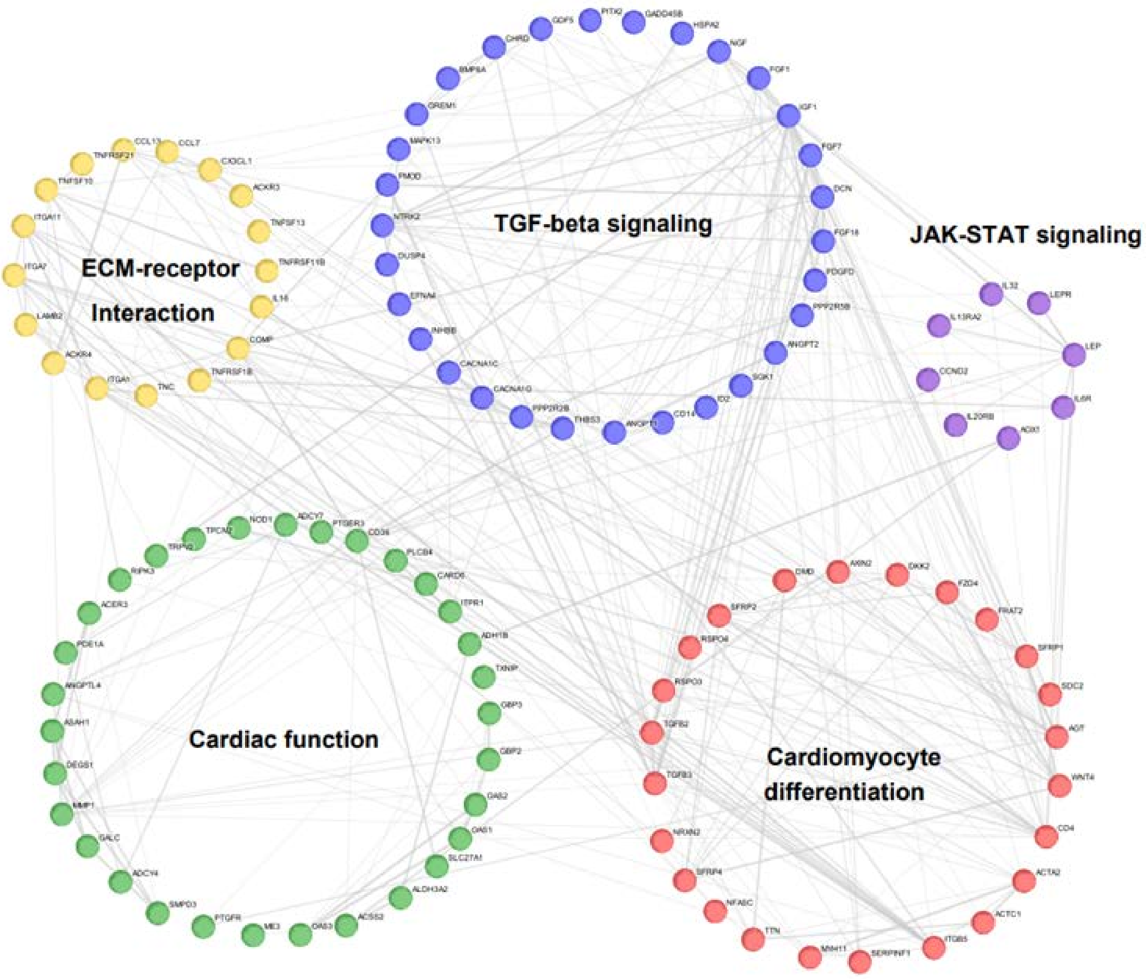

Supplementary Fig. 9. FA induces ECM-receptor interaction, TGF-beta signaling, and JAK-STAT3 signaling

The network analysis of KEGG pathway (fig. 6e)-related genes is shown based on protein-protein interactions derived from the STRING database.

3. SUPPLEMENTARY TABLES

Supplementary Table 1. Significantly upregulated GO terms

Upregulated Gene Ontology			
**MGTMM/Vehicle**			
**Category**	**Term**	**-log (P value)**	**Genes**
GO:0072359	circulatory system development	13.5892	CITED2, SRF, COL11A1, ELN, SERPINE1, SIX1, PTGS2, ACTG1, ROBO1, RGS4, RGS2, MDK, ENPP2, SLIT2, JARID2, JUNB, EGR1, SMAD3, ANXA2, GADD45A, UNC5B, FZD7, IRX3, CAV1, FN1, HMGA2, NR2F2, SULF1, TBX2, VEGFA, COL1A1, RGCC, COL1A2, COL5A1, DDIT3, ID1, COL8A1, TGFBI, PLCD3
GO:0048646	anatomical structure formation involved in morphogenesis	13.35276	FBN2, SDC4, CITED2, SRF, COL11A1, SERPINE1, SIX1, PTGS2, ACTG1, ROBO1, MDK, SLIT2, JUNB, TGM2, SMAD3, ITGA4, ANXA2, RPS7, GADD45A, UNC5B, TXNRD1, FZD7, IRX3, CAV1, FN1, HMGA2, LAMB1, SULF1, TBX2, VEGFA, COL1A1, KRT19, RGCC, COL5A1, ID1, COL8A1, TGFBI, PLCD3, KIF20B
GO:0072358	cardiovascular system development	13.05075	CITED2, SRF, SERPINE1, SIX1, PTGS2, ACTG1, ROBO1, MDK, ENPP2, SLIT2, JUNB, EGR1, ANXA2, GADD45A, UNC5B, CAV1, FN1, HMGA2, NR2F2, SULF1, TBX2, VEGFA, COL1A1, RGCC, COL1A2, COL5A1, DDIT3, ID1, COL8A1, TGFBI, PLCD3
GO:0001944	vasculature development	13.05075	CITED2, SRF, SERPINE1, SIX1, PTGS2, ACTG1, ROBO1, MDK, ENPP2, SLIT2, JUNB, EGR1, ANXA2, GADD45A, UNC5B, CAV1, FN1, HMGA2, NR2F2, SULF1, TBX2, VEGFA, COL1A1, RGCC, COL1A2, COL5A1, DDIT3, ID1, COL8A1, TGFBI, PLCD3
GO:0001568	blood vessel development	12.99337	CITED2, SRF, SERPINE1, SIX1, PTGS2, ACTG1, ROBO1, MDK, SLIT2, JUNB, EGR1, ANXA2, GADD45A, UNC5B, CAV1, FN1, HMGA2, NR2F2, SULF1, TBX2, VEGFA, COL1A1, RGCC, COL1A2, COL5A1, DDIT3, ID1, COL8A1, TGFBI, PLCD3
GO:0031589	cell-substrate adhesion	10.69856	SNED1, POSTN, SMAD3, SDC4, ITGA4, SRF, FZD7, SERPINE1, FN1, LAMB1, NID1, TRIOBP, ACTG1, VEGFA, COL1A1, MDK, PLAU, ID1, COL8A1, GBP1, S100A10
GO:0030198	extracellular matrix organization	10.54318	POSTN, SMAD3, ANXA2, ITGA4, COL11A1, ELN, CAV1, LAMB1, NID1, SULF1, COL1A1, ADAMTS2, RGCC, COL1A2, COL5A1, P4HA2, COL8A1, PTX3, TGFBI
GO:0048514	blood vessel morphogenesis	10.53702	CITED2, SRF, SERPINE1, SIX1, PTGS2, ACTG1, ROBO1, MDK, SLIT2, JUNB, ANXA2, GADD45A, UNC5B, CAV1, FN1, HMGA2, NR2F2, SULF1, TBX2, VEGFA, RGCC, ID1, COL8A1, TGFBI, PLCD3
GO:0043062	extracellular structure organization	10.51953	POSTN, SMAD3, ANXA2, ITGA4, COL11A1, ELN, CAV1, LAMB1, NID1, SULF1, COL1A1, ADAMTS2, RGCC, COL1A2, COL5A1, P4HA2, COL8A1, PTX3, TGFBI
GO:0016477	cell migration	10.13316	FBN2, SDC4, CITED2, SRF, CEP85L, SERPINE1, ATN1, SIX1, PTGS2, ACTG1, ROBO1, PLAU, MDK, ENPP2, SLIT2, IGFBP6, APPL2, POSTN, SMAD3, ITGA4, GADD45A, CAV1, FN1, NAV1, LAMB1, NR2F2, SULF1, VEGFA, PTP4A1, COL1A1, ASPM, VCAN, RGCC, NAV3, COL5A1, APEX1, MAP1B, DDIT3, ID1, ARHGEF2, KIF20B, PFN2
**MGTMM+FA/MGTMM**			
**Category**	**Term**	**-log (P value)**	**Genes**

GO:0009887	animal organ morphogenesis	16.88865	CSF3R, TNC, ADARB1, FGF1, AQP1, COMP, NDST1, FGF7, NAGLU, GJA5, HOXA3, HOXA7, PITX2, SOX4, HOXA4, CDON, TLE2, MSX2, IGFBP5, OSR1, DIO3, NPNT, SFRP4, OLFM1, SFRP1, SFRP2, HOXB9, HOXB4, HOXB3, HOXB8, HOXB7, HOXB6, HOXB5, CSF1R, DLX1, DLX2, SLC1A1, HTRA1, TACSTD2, TNFRSF11B, THY1, FOXO1, CHST11, ALX4, HOXC4, HOXC9, HOXC8, WNT4, TGFB2, JAG1, FZD5, TGFB3, ESR1, MAFB, ID2, FGF18, TMEM119, CPE, ALPL, FAT3, HOXD4, SDK2, THRB, TENM3, CTSZ, SLC40A1, LAMC2, SNX10, PKD2, SMPD3, ADAMTS5, SIX2, RSPO3, CEMP1, CD34, DSP, HEG1, NOG, PAX3, NRG1, AXIN2, IGFN1, DCN, ACTA2, GREM1, ACTA1, ALDH1A3, MFRP, HAND2, FAM20A, ATP6V1B1, TUFT1, GREB1L, LRP5, LRP4, NTN1, NKD1, TTN, THBS3, ACAN, FRZB, GSC, TNNI1, HAS2, MSX1, IRX1, TBX1, WWOX, NTRK2, PRRX1, LAMB3, LAMB2, TMEM176B, CFLAR, TBX19, VAX2, TSHR, AGT, MFAP5, HEYL, ACTC1, ASB2
GO:0001501	skeletal system development	14.54272	HEXB, HEXA, SNX10, HAPLN3, COMP, SMPD3, NDST1, CTSK, SIX2, HOXA3, COL10A1, HOXA7, PITX2, SOX4, HOXA4, ADAMTS7, CMKLR1, MSX2, SLC2A10, OSR1, NOG, EN1, AXIN2, RUNX3, GREM1, SFRP4, SFRP1, HOXB9, SFRP2, MAF, HAND2, HOXB4, HOXB3, HOXB8, HOXB7, HOXB6, HOXB5, DLX1, DLX2, CHRD, LRP5, PRELP, TNFRSF11B, COL19A1, THBS3, ACAN, EXTL1, SCIN, CHST11, FRZB, GSC, HOXC5, HOXC4, HAS2, ALX4, CYTL1, MSX1, HOXC9, HOXC8, HOXC6, TBX1, WWOX, TGFB2, PRRX1, TGFB3, LUM, BMP8A, IGF1, GDF5, LEP, ZNF219, FGF18, TMEM119, ALPL, HOXD4
GO:0030198	extracellular matrix organization	10.35825	VIT, OLFML2B, ECM2, DPT, TNFRSF11B, SPINT2, ADAMTS10, COL19A1, DPP4, SMPD3, COMP, ADAMTS5, ACAN, MMP24, ADAMTSL4, CTSK, HAS3, CYP1B1, COL10A1, HAS2, MYH11, SLC39A8, ELANE, ADAMTS7, TGFB2, LAMB3, COL24A1, MIA, LAMB2, MMP1, LUM, COL23A1, CFLAR, MMP8, TNFRSF1B, NPNT, AGT, GREM1, SMOC2, MYO1E, CCDC80, SFRP2, CRISPLD2, COL4A4, COL6A6, FMOD, PHLDB2
GO:0022610	biological adhesion	10.35214	VIT, SPON2, SPON1, CSF3R, CLSTN2, TNC, SPINT2, IL1RAP, COMP, ISLR, CYP1B1, NRCAM, SLC39A8, HOXA7, KIFAP3, IL6R, SOX4, EPHB3, CD177, CDON, CD93, EPDR1, ATP1B1, NPNT, RUNX3, SFRP1, SFRP2, CCDC80, ADAM12, MYL9, PHLDB2, FBLN7, CHRD, TACSTD2, DPT, IL20RB, THY1, RASGRP1, DPP4, CLCA2, PLXNA2, MBP, APOD, PLXNA4, ELANE, WNT4, HLA-DRB5, TGFB2, CADM4, JAG1, TNFSF14, FZD4, MCAM, INHBB, IGF1, DAB1, PCDHB2, ITGA11, TNFSF9, HLA-DRA, GNRH1, FAT3, SDK2, HLA-DRB1, ADA, PCDHB7, LGALS3BP, TENM2, ECM2, TENM3, ITGB5, ITGB2, LAMC2, LRRC32, HAPLN3, CX3CL1, TMEM47, MMP24, C1QTNF1, ME3, EMB, CD36, CD34, HLA-DPA1, DSP, BCAS3, RIPK3, ITGA1, NRG1, TNFRSF1B, IGFN1, GREM1, APBB1IP, CLDN15, CDHR3, ITGA7, COL6A6, ZP3, TNFRSF21, LRRC15, CSTA, AMIGO1, NRXN2, NTN1, COL19A1, THBS3, ACAN, HLA-DMA, HLA-DMB, SRPX2, HAS2, DMD, PCDH1, JAM2, IL32, FARP2, AOC3, CD74, ANGPT2, ANGPT1, LAMB3, CDKN2A, LAMB2, CD70, MYBPH, LAPTM5, PCDHB14, PCDHB13, PCDHB10, XG, NFASC, CD4, LEP, PCDHB16, HLA-DPB1, ACKR3, CCL28, ADGRL3
GO:0072358	cardiovascular system development	10.13041	HSPB6, ITGB2, CLEC14A, PRL, PKD2, FGF1, MEOX2, CX3CL1, AQP1, COMP, NDST1, BMPER, GJA5, GPER1, HOXA3, ENPP2, LEPR, RSPO3, CYP1B1, NRCAM, HOXA7, VSTM4, CD34, IL6R, SOX4, EPHB3, BCAS3, CCBE1, MYOCD, SLC2A10, HEG1, SERPINF1, OSR1, NOG, SERPINB7, DCN, ACTA2, GREM1, SFRP1, SFRP2, DDAH1, TNMD, HAND2, ADAM12, HOXB3, ANGPTL2, ANGPTL4, SCG2, RAPGEF3, NOTCH3, EPAS1, SLC1A1, LRP5, THY1, FOXO4,

			FOXO1, APELA, SRPX2, TMEM204, PDGFD, HAS2, APOD, APOE, WNT4, TBX1, NTRK2, TGFB2, ANGPT2, JAG1, PRRX1, ANGPT1, FZD5, FZD4, MCAM, C1GALT1, NFATC2, CFLAR, SOD2, PTPN14, PLXDC1, AGT, APLN, MYO1E, SMOC2, LEP, FGF18, ACKR3
GO:0001944	vasculature development	10.13041	HSPB6, ITGB2, CLEC14A, PRL, PKD2, FGF1, MEOX2, CX3CL1, AQP1, COMP, NDST1, BMPER, GJA5, GPER1, HOXA3, ENPP2, LEPR, RSPO3, CYP1B1, NRCAM, HOXA7, VSTM4, CD34, IL6R, SOX4, EPHB3, BCAS3, CCBE1, MYOCD, SLC2A10, HEG1, SERPINF1, OSR1, NOG, SERPINB7, DCN, ACTA2, GREM1, SFRP1, SFRP2, DDAH1, TNMD, HAND2, ADAM12, HOXB3, ANGPTL2, ANGPTL4, SCG2, RAPGEF3, NOTCH3, EPAS1, SLC1A1, LRP5, THY1, FOXO4, FOXO1, APELA, SRPX2, TMEM204, PDGFD, HAS2, APOD, APOE, WNT4, TBX1, NTRK2, TGFB2, ANGPT2, JAG1, PRRX1, ANGPT1, FZD5, FZD4, MCAM, C1GALT1, NFATC2, CFLAR, SOD2, PTPN14, PLXDC1, AGT, APLN, MYO1E, SMOC2, LEP, FGF18, ACKR3
GO:0016477	cell migration	9.772093	IFITM1, CSF3R, ADARB1, FGF1, AQP1, FGF7, BMPER, ENPP2, ADORA1, CYP1B1, NRCAM, HOXA7, VSTM4, PITX2, IL6R, EPHB3, CD177, CMKLR1, SEMA6C, CCBE1, S100A2, MSX2, IGFBP5, SERPINF1, SEMA6D, SAP30, SFRP1, SFRP2, HOXB9, SCG2, PHLDB2, CSF1R, SDC2, CHRD, TACSTD2, THY1, FOXO4, CYP19A1, MST1L, DPP4, APELA, PDGFD, PLXNA2, APOD, APOE, SRGAP3, PPARGC1A, CCR10, ELANE, WNT4, TGFB2, JAG1, TNFSF14, FZD4, RARRES2, MCAM, IDH2, NFATC2, IGF1, BST2, IGSF10, DAB1, BAMBI, ITGA11, FGF18, NOX4, GNRH1, FAT3, ADA, ITGB5, HEXB, IL24, ITGB2, LAMC2, CLEC14A, MEOX2, CX3CL1, SMPD3, GPER1, SIX2, LBP, CD34, BCAS3, MYOCD, RIPK3, NOG, ITGA1, RRAS2, NRG1, IL16, POMGNT2, DCN, ADRA2A, GREM1, HAND2, ITGA7, ZP3, CCL13, LRRC15, SYNPO2, LRP5, PRR5L, SEMA3F, NTN1, CLN3, P2RY6, ACVR1C, SRPX2, CCL8, CCL7, MGAT5, HAS2, LURAP1, JAM2, TBX1, NTRK2, CD74, ANGPT2, ANGPT1, LAMB3, LAMB2, SOD2, SLC8B1, AGT, XG, SMOC2, FMNL1, LEP, ACKR4, ACKR3, ASB2, CCL28, MDGA1, MIXL1, CCL26, ADGRL3
GO:0042127	regulation of cell proliferation	9.633287	IFITM1, TNC, ADARB1, SPINT2, FGF1, AQP1, COMP, FGF7, CCND2, RASSF5, HOXA3, ADORA1, CYP1B1, PTGDS, KIFAP3, IL6R, SOX4, CDON, KRT4, MSX2, IGFBP5, SERPINF1, OSR1, RUNX3, SFRP4, ALDH3A1, SFRP1, SFRP2, TXNIP, SCG2, PTGFR, CSF1R, NOTCH3, PDE1A, HTRA1, CHRD, TACSTD2, DPT, IL20RB, FOXO4, MST1L, FOXO1, DPP4, APELA, GJC2, SCIN, CHST11, PDGFD, APOD, APOE, PPARGC1A, ELANE, TGFB2, NMB, CADM4, JAG1, FZD5, TGFB3, TNFSF13, IDH2, NFATC2, IGF1, GDF5, ESR1, FOSL2, PARP10, BST2, BAMBI, ID2, ACER3, FGF18, TMEM119, TNFSF9, NOX4, GNRH1, COPS8, HLA-DRB1, ADA, IL24, CTSZ, AKR1B1, LAMC2, PRL, LRRC32, PKD2, HAPLN3, CX3CL1, SMPD3, LBH, GPER1, SIX2, LDLRAP1, HLA-DPA1, PTGIR, MYOCD, RIPK3, NOG, ITGA1, NRG1, AVPR1A, AXIN2, SERPINB7, NGF, TNFRSF1B, ADRA2A, GREM1, DDAH1, MYOD1, TNMD, HAND2, SGK1, ZP3, TNFRSF21, LRP5, CRIP2, NTN1, SLC25A27, P2RY6, HLA-DMB, ACVR1C, CCL8, CLMN, FRZB, ZNF503, HAS2, DMD, MSX1, TP53I11, TBX1, NTRK2, CD74, PRRX1, ANGPT1, CDKN2A, CD70, AKR1C3, AKR1C2, CFLAR, SOD2, TBX19, PTPN14, EPOR, TSHR, AGT, APLN, SST, LEP, KLF9, HLA-DPB1, ACKR3, KCNK2, CCL26
GO:0048468	cell development	9.514502	NRK, TNC, STMN2, ADARB1, SPINT2, COMP, PREX2, RIMS2, RNF112, NAGLU, KIF5A, CAPN3, NRCAM, KIFAP3, PITX2, SOX4, EPHB3, CDON, SEMA6C, MSX2, SERPINF1, SEMA6D, OSR1, NANOS3, DIO3, SYPL2, RUNX3, OLFM1, SFRP1, SFRP2, MAF, HECW1, HOXB3, KCTD11, MGARP, MYL9, CSF1R, DLX1,

			NOTCH3, DLX2, NRN1, ARHGEF25, EPAS1, SDC2, TACSTD2, THY1, EFNA4, GMCL1, GJC2, BRINP1, CHST11, TTPA, PLXNA2, MAP6, MYH11, MBP, APOD, APOE, PLXNA4, EXPH5, WNT4, GSTM3, TGFB2, JAG1, FZD5, SYT1, FZD4, CCDC136, IDH2, TRPV2, NFATC2, INHBB, NBEAL2, IGF1, HSPA2, GDF5, ESR1, FOSL2, IGSF10, DAB1, CABYR, ID2, FGF18, GNRH1, FAT3, TDRD10, SERPINI1, PIWIL2, UCN, TENM2, THRB, TENM3, HEXB, HEXA, CTSZ, AKR1B1, LAMC2, LDB3, CX3CL1, SMPD3, ADAMTSL4, GPER1, KIF13B, EFHD1, EMB, GPR37, STRBP, HEG1, NOG, ITGA1, PPP2R5B, RRAS2, EN1, NRG1, RTN4RL2, AXIN2, BPGM, NGF, TNFRSF1B, ACTA2, ACTA1, GPRC5B, MFRP, RTN4RL1, MYOD1, TNMD, HAND2, B3GNT2, SGK1, ZP3, B4GALT6, TNFRSF21, RAPGEF3, B4GALT5, ATL1, AMIGO1, CDNF, LRP5, LRP4, AKAP6, SEMA3F, GDPD5, AURKC, NTN1, POMZP3, TTN, ACAN, PARD6B, CLMN, FRZB, CHN1, RAB29, HAS2, DMD, JAM2, IRX1, FARP2, TBX1, NTRK2, ANGPT2, PRRX1, LAMB3, LAMB2, IRX6, LGI4, C1GALT1, NAP1L2, SORBS2, CFLAR, SOD2, VAX2, TSHR, AGT, MYO1E, HEYL, NFASC, DPY19L2, ACTC1, ZGLP1, LEP, ASB2
GO:0003002	regionalization	9.492543	MEOX2, SIX2, HOXA3, HOXA7, PITX2, HOXA4, CDON, MSX2, OSR1, NOG, PIFO, EN1, AXIN2, GREM1, SFRP1, HOXB9, SFRP2, HOXB4, HOXB3, HOXB8, HOXB7, HOXB6, HOXB5, DLX1, DLX2, CHRD, LRP5, LRP4, SEMA3F, NKD1, GSC, HOXC5, PLXNA2, HOXC4, ALX4, MSX1, HOXC9, HOXC8, HOXC6, IRX1, TBX1, FZD5, FBXL15, TBX19, VAX2, HEYL, MAFB, HOXD4, TDRD10

Supplementary Table 2. Significantly upregulated KEGG pathways

Upregulated KEGG pathway			
**MGTMM/Vehicle**			
**Category**	**Term**	**-log (P value)**	**Genes**
hsa03010	Ribosome	4.462167	RPL4, RPL21, RPS7, RPL31, RPL34, RPL11, RPL36, RPL14, RPSA, RPL23A, RPL26
hsa05171	Coronavirus disease - COVID-19	3.909574	RPL4, RPL21, RPS7, RPL31, RPL34, RPL11, RPL36, RPL14, RPSA, RPL23A, FOS, RPL26
hsa04933	AGE-RAGE signaling pathway in diabetic complications	3.661233	COL1A1, EGR1, SMAD3, COL1A2, SERPINE1, FN1, PLCD3, VEGFA
hsa00270	Cysteine and methionine metabolism	3.416045	MAT2A, GOT1, PSAT1, SMS, PHGDH, BCAT1
hsa01230	Biosynthesis of amino acids	2.650934	MAT2A, GOT1, PSAT1, ASNS, PHGDH, BCAT1
hsa05205	Proteoglycans in cancer	2.435694	COL1A1, COL1A2, SDC4, PLAU, FZD7, CAV1, FN1, ACTG1, VEGFA
hsa04512	ECM-receptor interaction	2.34943	COL1A1, COL1A2, SDC4, ITGA4, FN1, LAMB1
hsa05418	Fluid shear stress and atherosclerosis	2.141927	SDC4, CAV1, FOS, ARHGEF2, TXN, ACTG1, VEGFA
hsa04974	Protein digestion and absorption	2.06289	COL1A1, COL1A2, COL5A1, COL11A1, ELN, COL8A1
hsa05132	Salmonella infection	1.940909	TUBA1C, TUBA1B, ANXA2, ARPC1A, FOS, TXN, PFN2, ACTG1, S100A10
hsa04510	Focal adhesion	1.928585	COL1A1, COL1A2, ITGA4, CAV1, FN1, LAMB1, ACTG1, VEGFA
hsa04210	Apoptosis	1.585857	TUBA1C, TUBA1B, GADD45A, DDIT3, FOS, ACTG1
hsa05130	Pathogenic Escherichia coli infection	1.46452	CLDN11, TUBA1C, TUBA1B, ARPC1A, FOS, ARHGEF2, ACTG1
hsa04390	Hippo signaling pathway	1.356189	SMAD3, FZD7, ID1, SERPINE1, CTGF, ACTG1
hsa00970	Aminoacyl-tRNA biosynthesis	1.323813	YARS, WARS, SARS, IARS
**MGTMM+FA/MGTMM**			
**Category**	**Term**	**-log (P value)**	**Genes**
hsa05414	Dilated cardiomyopathy	2.02977	TGFB2, DTNA, ITGB5, TGFB3, ITGA1, ADCY4, CACNA1C, IGF1, ADCY7, AGT, TTN, ACTC1, ITGA11, ITGA7, DMD
hsa04658	Th1 and Th2 cell differentiation	1.759134	NOTCH3, HLA-DRB5, JAG1, RUNX3, MAPK13, HLA-DMA, CD4, MAF, HLA-DPB1, HLA-DRA, MAML3, HLA-DRB1, HLA-DPA1
hsa05410	Hypertrophic cardiomyopathy	1.759134	TGFB2, DTNA, ITGB5, TGFB3, ITGA1, CACNA1C, IGF1, AGT, TTN, ACTC1, ITGA11, ITGA7, DMD
hsa04060	Cytokine-cytokine receptor interaction	3.38295	CCL13, IL20RB, TNFRSF11B, IL1RAP, CX3CL1, CCL7, TNFSF10, LEPR, IL13RA2, IL6R, IL32, TGFB2, TGFB3, TNFSF13, BMP8A, IL16, INHBB, NGF, TNFRSF1B, GDF5, CD4, LEP, ACKR4, ACKR3, TNFRSF21
hsa05323	Rheumatoid arthritis	1.623816	TGFB2, HLA-DMA, HLA-DRB5, ANGPT1, TGFB3, MMP1, CTSK, TNFSF13, HLA-DPB1, HLA-DRA, HLA-DRB1, HLA-DPA1

hsa04142	Lysosome	1.894452	ASAH1, HEXB, HEXA, CTSZ, CLTB, GALC, CLN3, NAGLU, CTSK, SLC17A5, AGA, CTSF, PPT2, CTSD
hsa01100	Metabolic pathways	11.23139	B4GALT3, CHPF, GALNT16, GALNT15, HEXB, HEXA, QPRT, AKR1B1, HNMT, ENO2, GLS, SMPD3, NDST1, STS, NAGLU, NMRK1, ENPP2, AOX1, ME3, A4GALT, PTGDS, GPX3, BHMT2, BPGM, POMGNT2, INMT, PGM2L1, ALDH3A2, ALDH1A3, BTD, PLCB4, CYP2U1, NME7, ETHE1, CHDH, B3GNT2, RDH5, ACOT1, HAGH, ST6GALNAC6, B4GALT5, ASAH1, ACSS2, NNMT, ADH1B, PDE1A, AK1, GDPD1, ADCY4, GSTT2, GSTT1, AK4, ADCY7, FGGY, MGAT5, DSEL, PRDM16, MAN1C1, ST3GAL5, CKB, CA12, AOC3, GSTM3, GALNT5, PTGES2, AKR1C1, C1GALT1, IDH2, AKR1C3, FMO1, AKR1C2, FMO3, AKR1C4, DHODH, GALC, NMNAT1, ACER3, ALPL, DEGS1, PPT2, PDE7B, ADA, GSTM5
hsa05330	Allograft rejection	0.947226	HLA-DMA, HLA-DRB5, HLA-DPB1, HLA-DRA, HLA-F, HLA-DRB1, HLA-DPA1
hsa04612	Antigen processing and presentation	1.35318	CD74, HLA-DMA, HLA-DRB5, CD4, HLA-DPB1, HLA-DRA, HSPA2, HLA-F, HLA-DRB1, HLA-DPA1
hsa04350	TGF-beta signaling pathway	1.623816	GREM1, TGFB2, TGFB3, ID2, CHRD, BMP8A, INHBB, LRRC32, FMOD, PITX2, GDF5, DCN

Supplementary Table 3. qRT-PCR primer list

Species	Gene	Forward primer (5'-3')	Reverse primer (5'-3')	Product (bp)
Mouse	Atp2a2	TTCTGCTTATCTTGGTAGCCAA	CTTTCTGTCCTGTCGATACACT	125
	Atp2b1	AACGACTGGAGCAAGGAGAA	CCGTACTTCACTTGGGCAAT	146
	Cav3	GCTCGGATCATCAAGGACAT	ACACCGTCGAAGCTGTAGGT	153
	Gapdh	TTCACCACCATGGAGAAGGC	GGCATGGACTGTGGTCATGA	236
	Jph2	GCTGGCTCCAGACTTCTACC	GGCTCCAGCAAGCTCTCA	93
	Kcnh2	GTGCTGCCTGAGTATAAGCTG	CCGAGTACGGTGTGAAGACT	136
	Kcnj2	CACTTCCACTCCATGTCCCC	GGAGAGATGGATGCTTCCGAG	127
	Mcu	AAAGGAGCCAAAAAGTCACG	AACGGCGTGAGTTACAAACA	200
	Micu1	AAGAACACTCCCTGCCATTT	GCCAGGGTCATCTGCATTAT	107
	Micu2	TGGAGCACGACGGAGAGTAT	GCCAGCTTCTTGACCAGTGT	97
	Myl2	GGACACATTTGCTGCCCTA	ATCGTGAGGAACACGGTGA	105
	Myl7	CAGACCTGAAGGAGACCT	CTGTGGTTCAGGGCTCAGTC	110
	Ncx1	CGAGACTGTGTCGAACCTGA	TCAGGGACCACGTAAACACA	183
	Nfe2l2	CTGAACTCCTGGACGGGACTA	CGGTGGGTCTCCGTAAATGG	182
	Nrf1	GCACCTTTGGAGAATGTGGT	CTGAGCCTGGGTCATTTTGT	165
	Pln	AAAGTGCAATACCTCACTCGC	GGCATTTCAATAGTGGAGGCTC	56
	Ppargc1a	GAATCAAGCCACTACAGACACCG	CACGAAAGGCTCAAGAGGGATG	136
	Ryr2	ACCTACTCCGAAGGCTGGTGTT	TTCTTCCGAGGCAGCACCAAAG	149
	Sirt1	CGGCTACCGAGGTCCATATAC	CAGCTCAGGTGGAGGAATTGT	109
	Sirt3	GGATTCGGATGGCGCTTGA	CACCTGTAACACTCCCGGAC	116
	Tfam	CACCCAGATGCAAAACTTTCAG	CTGCTCTTTATACTTGCTCACAG	147
	Tnnt2	GGCTCACTTCGAGAACAGGA	TCATTGCGAATACGCTGCT	108
Human	BIN1	AACTAACCTGCTCCGAAATCAG	GTAGAAACCTACGCGGCTGT	124
	BMP2	CAGACCACCGGTTGGAGA	CCCACTCGTTTCTGGTAGTTCT	95
	CACNA1C	GTGGAGTCGTCCTGGTTGT	AACCATACACCCCACGACAT	152
	CAV3	GAGGCCCAGATCGTCAAG	TCACGTCTTCAAAATCCACCT	106
	CD36	AAATCTGGGCTTTGGATGTG	GAGCAATTGCAAGTGTCCAA	171
	CPT1B	TGTGAGTGACTGGTGGGAAG	TTGATGAGCACAAGGTCCAT	96
	DES	GTACCAGGACCTGCTCAACG	GGTCTGGATGGGGAGATTG	106
	GAPDH	GAGTCCACTGGCGTCTTCAC	TTCACACCCATGACGAACAT	119
	GATA4	TCCCTCTTCCCTCCTCAAAT	TCAGCGTGTAAAGGCATCTG	194
	GDF5	GAAAGGGAGGCAACAGCA	ACGGGACCTCGGTCATCT	94
	ITGA5	CCCATTGAATTTGACAGCAA	TGCAAGGACTTGTACTCCACA	92
	ITGA6	TTTGAAGATGGGCCTTATGAA	CCCTGAGTCCAAAGAAAAACC	102
	ITGA7	TCCCTGCCAACAGCTACTTT	CCACAGCACCCTTGTGGT	114
	ITGAV	GCACCCTCCTTCTGATCCT	GAGGACCTGCCCTCCTTC	113
	ITGB1	CGATGCCATCATGCAAGT	AGTGAAACCCGGCATCTG	95
	ITGB3	GCCCTGCTCATCTGGAAAC	TACAGTGGGTTGTTGGCTGT	110
	JPH2	GAGGTGGAGGTGGAAGAGG	TCAGGTCAGGAGGTGAACAA	99
	KCNH2	CAACCTGGGCGACCAGATAG	GGTGTTGGGAGAGACGTTGC	142

	KCNJ2	TTCAGTCACAATGCCGTGATT	GCTTTTCCGAAGATTGCCCA	78
	MEF2C	ATCTGCCCTCAGTCAGTTGG	CTGACTTGATGTTGAGGCTTTG	98
	MESP1	CCGAGTCCTGGATGCTCTC	AGTCTGGGGACGAGACGAG	96
	MYL7	GGGTGGTGAACAAGGATGAG	GTGTCAGGGCGAACATCTG	93
	MYL2	GCAGGCGGAGAGGTTTTC	AGTTGCCAGTCACGTCAGG	74
	mtDNA	CACCCAAGAACAGGGTTTGT	TGGCCATGGGTATGTTGTTA	107
	MYH6	ATGAACGCGGAGCTCACT	CAGGTCATCAATGTCCTTCTTG	78
	MYH7	GCTGCAGAAGAAGCTCAAGG	AGGTCTGAGCGCAGCTTCT	108
	MYOCD	TCACTTTCTGCCCTCATCCT	TCGTGTGCTCCTGAGTTCTG	102
	nDNA	TGCTGTCTCCATGTTTGATGTATCT	TCTCTGCTCCCCACCTCTAAGT	86
	PPARGC1A	CATCAGCAATGGATGAGACC	CCAACCAGAGCAGCACACT	94
	PPARA	TGAGGGGGTAACAGCAAATC	GCTAACTGCAGAGGGTGAGG	244
	ROCK1	GGGGACAGTTTTGAGACTCG	AAAGCATCCAATCCATCCAG	101
	ROCK2	ATAGCCCCTGGGTGGTTC	CCATTTTTCAGGCACATCATAA	128
	RYR2	CCTTTCTCCATCCCTTCCTC	CAAAGCAGTCCCTTTTCAGC	240
	SIRT1	TGTACGACGAAGACGACGAC	TTCATCACCGAACAGAAGGTT	95
	TLN1	ACCAGTGACTATGGCCGTCT	CGGTGTTTGATATGGGAACC	89
	TLN2	CGGTGTTTGATATGGGAACC	CGGTGTTTGATATGGGAACC	77
	TBX18	CCTGGAATTCCCAAGCAAG	GAGGAGCCAGACAAAAGGTG	101
	TBX5	CCAGGAGCATAGCCAAATTTAC	AGGGCTTCTTATAGGGATGGTC	83
	TFAM	GTTTCTCCGAAGCATGTGG	AGATGAAAACCACCTCGGTAAA	127
	TGFB1	AGTGGTTGAGCCGTGGAG	TGCAGTGTGTTATCCCTGCT	70
	TGFB2	CCTTCTTCCCCTCCGAAAC	AGAGCACCTGGGACTGTCTG	82
	TGFBR1	GCAGACTTAGGACTGGCAGTAAG	AGAACTTCAGGGGCCATGT	104
	TGFBR2	CACCGCACGTTCAGAAGTC	TGGATGGGCAGTCCTATTACA	86
	TNNI3	GCAGATGCCATGATGCAG	CACCTCCCGGTTTTCCTT	114
	TNNT2	ACCAGGGCAGAAGAAGATGA	TTGGTTTGGACTCCTCCATT	76
	VCL	GATGAAGCTCGCAAATGGTC	TCTGCCTCAGCTACAACACCT	77

Supplementary Table 4. List of primary and secondary antibodies used in this study

Name of antibody	Company	Cat. No.	Host animals	Dilutions	
				WB	IF
**Primary antibody**					
ACTN	Sigma-Aldrich	A7811	Mouse		1:400
CAV3	Abcam	ab2912	Rabbit	1:1000	
cJun	Cell Signaling Technology	9165	Rabbit	1:1000	
COL1A1	Cell Signaling Technology	66948	Mouse	1:1000	
CPT1B	Proteintech, Rosemont, IL, USA	22170-1-AP	Rabbit	1:1000	
DAPI	Sigma-Aldrich	D9542			1 μg/mL
ERK1/2	Cell Signaling Technology	9102	Mouse	1:1000	
FN1	Cell Signaling Technology	26836	Rabbit	1:1000	
GAPDH	Sigma-Aldrich	G8795	Mouse	1:2000	
GSK3B	Cell Signaling Technology	9315	Rabbit	1:1000	
H3K27me3	Abcam	ab6002	Mouse		1:400
H3K4me3	Abcam	ab8580	Rabbit	1:1000	1:400
Hoechst	Invitrogen	H21492			1 μg/mL
JAK2	Cell Signaling Technology	3230	Rabbit	1:1000	
JPH2	Thermo Fisher Scientific	40–5300	Rabbit	1:1000	1:200
Laminin	Invitrogen	PA1-16730	Rabbit	1:1000	
MYL2	Proteintech	PTG10906-1-AP	Rabbit	1:1000	
MYL7	Synaptic Systems, Goettingen, Germany	311011	Mouse	1:1000	
p21	Cell Signaling Technology	2947	Rabbit	1:1000	
p-cJun	Cell Signaling Technology	2361	Rabbit	1:1000	
p-ERK1/2	Cell Signaling Technology	9106	Rabbit	1:1000	
p-JAK2	Cell Signaling Technology	3771	Rabbit	1:1000	
PPARGC1A	NOVUS Biologicals, Littleton, CO, USA	NBP1 04676	Rabbit	1:1000	
p-SMAD1/5	Cell Signaling Technology	9516	Rabbit	1:1000	
p-SMAD2	Cell Signaling Technology	3108	Rabbit	1:1000	
p-SMAD3	Cell Signaling Technology	9520	Rabbit	1:1000	
p-STAT3	Cell Signaling Technology	9131	Rabbit	1:1000	
SMAD1	Cell Signaling Technology	6944	Rabbit	1:1000	
SMAD2	Cell Signaling Technology	3122	Rabbit	1:1000	
SMAD3	Cell Signaling Technology	9523	Rabbit	1:1000	
SMAD5	Cell Signaling Technology	9517	Rabbit	1:1000	
STAT3	Cell Signaling Technology	9132	Rabbit	1:1000	
TFAM	Abcam	ab131607	Rabbit	1:1000	
TNNI3	Abcam	ab10231	Mouse		1:400
TNNT2	Thermo Fisher Scientific	MA5-12960	Mouse	1:1000	1:200
**Secondary antibody**					
Anti-mouse HRP	Cell Signaling Technology	7076		1:3000	
Anti-rabbit HRP	Cell Signaling Technology	7074		1:3000	

Alexa Fluor 488 anti-mouse	Molecular Probes	A11001	Goat		1:1000
Alexa Fluor 555 anti-rabbit	Invitrogen	A32732	Goat		1:1000
Alexa Fluor 594 anti-mouse	Molecular Probes	A11005	Goat		1:1000
Alexa Fluor 594 anti-rabbit	Molecular Probes	A21442	Chicken		1:1000
Alexa Fluor 647 anti-mouse	Invitrogen	A32728	Goat		1:1000
